# Development and Evaluation of a Physiologically Based Pharmacokinetic Model for Cipepofol Across Diverse Clinical Populations

**DOI:** 10.3390/pharmaceutics18060763

**Published:** 2026-06-22

**Authors:** Junmin Li, Longjie Li, Fangbin Ding, Meixia Chen, Mengyue Hu, Xiaoqiang Xiang, Jing Tang

**Affiliations:** 1 Obstetrics & Gynecology Hospital of Fudan University, Shanghai Key Lab of Reproduction and Development, Shanghai Key Lab of Female Reproductive Endocrine Related Diseases, Shanghai 200433, China; 22111030044@m.fudan.edu.cn; 2 Department of Clinical Pharmacy and Pharmacy Administration, School of Pharmaceutical Sciences, Fudan University, Shanghai 201203, China; 23211030010@m.fudan.edu.cn (L.L.); 25111030034@m.fudan.edu.cn (F.D.); 3 Haisco Pharmaceutical Group Co., Ltd., Chengdu 856000, China; chenmeixia@haisco.com (M.C.); humy@haisco.com (M.H.); 4 Quzhou Fudan Institute, Quzhou 324003, China; 5 State Key Laboratory of Advanced Drug Formulations for Overcoming Delivery Barriers, Shanghai 201203, China

**Keywords:** cipepofol, physiologically based pharmacokinetic modeling, special populations, individualized simulation

## Abstract

**Background/Objectives**: Cipepofol is a novel intravenous anesthetic whose pharmacokinetics (PK) may vary with dosing regimens, sampling sites, and physiological differences across populations. However, clinical PK data remain fragmented across study settings and are limited for special populations and individualized perioperative use, highlighting the need for a mechanistic modeling framework. This study aimed to develop and evaluate a physiologically based pharmacokinetic (PBPK) model for cipepofol across diverse populations. **Methods**: Clinical data from nine studies were included, comprising 371 subjects and 3521 plasma concentration measurements. The model was established in healthy adults using HSK3486-101, qualified using healthy-adult data from HSK3486-111 and anesthesia induction datasets, and extrapolated to hepatic impairment, renal impairment, and elderly populations using pathophysiology-informed adjustments. Individualized external validation was further performed in adult and pediatric surgical patients using actual clinical dosing histories. Model performance was evaluated using concentration–time profiles, goodness-of-fit plots, fold error, and geometric mean fold error (GMFE) for C_max_ and AUC_0–t_. **Results**: The model adequately described both arterial and venous plasma concentration–time profiles across the establishment, qualification, extrapolation, and external validation datasets. Most predicted concentrations were within two-fold of the observed values, and the overall GMFE values were 1.22 for C_max_ and 1.21 for AUC_0–t_. Simulated exposure differences in hepatic impairment, renal impairment, and elderly subjects were generally limited, suggesting no clinically meaningful PK changes from a PK exposure perspective in these populations. The model also reproduced arterial–venous concentration differences and supported the major contributions of UGT1A9 and CYP2B6 to cipepofol clearance. **Conclusions**: This PBPK model provides a mechanistic framework for characterizing cipepofol disposition and may inform future model-informed dosing studies.

## 1. Introduction

Cipepofol (formerly known as ciprofol, HSK3486) is a novel 2,6-disubstituted phenol intravenous anesthetic developed as an alternative to propofol for procedural sedation and general anesthesia [1]. Recent randomized clinical trials in adults undergoing elective surgery have shown that cipepofol provides non-inferior efficacy to propofol for anesthesia induction and maintenance, while markedly reducing the incidence of injection-site pain [2]. In addition, recent systematic reviews and meta-analyses have supported its comparable anesthetic efficacy and suggested potential safety advantages over propofol, particularly with respect to injection pain and, in some analyses, hypotension [3,4]. These findings support cipepofol as a clinically relevant anesthetic agent for which a thorough understanding of pharmacokinetics (PK) and disposition is important to guide clinical use.

However, characterizing cipepofol PK in perioperative settings remains challenging. In clinical anesthesia, drug administration is highly dynamic and typically involves induction boluses, maintenance infusions, and repeated dose adjustments according to surgical stimulation and anesthetic depth. Moreover, after intravenous administration, early concentration–time profiles may differ substantially between arterial and venous sampling sites, complicating the interpretation of observed plasma concentrations [5,6]. This challenge is possibly further compounded in special populations, such as patients with hepatic impairment, renal impairment, older adults, and children, for whom dedicated clinical data are often limited [7,8,9]. Physiologically based pharmacokinetic (PBPK) modeling provides a mechanistic framework that integrates drug-specific properties with system-specific physiology, and is widely recognized as a valuable tool for exposure prediction, dose extrapolation, and model-informed drug development in special populations [10,11,12].

The currently available evidence provides a strong foundation for PBPK modeling of cipepofol. Mechanistic metabolism studies have demonstrated that cipepofol clearance in humans is mediated predominantly by UDP-glucuronosyltransferase (UGT) pathways, especially UGT1A9, with cytochrome P450 2B6 (CYP2B6) also making a substantial contribution, and with both hepatic and extrahepatic metabolism involved [13]. In parallel, population pharmacokinetics/pharmacodynamics (PK/PD) and exposure-response analyses have shown that body weight, age and blood sampling site significantly influence cipepofol PK in patients undergoing elective surgery [14]. A mechanistic cipepofol PBPK model has also been reported previously to inform dose decisions in specific populations [15]. However, the available clinical and modeling evidence remains largely population-specific rather than mechanistically integrated. A unified PBPK framework that integrates healthy-adult model establishment, perioperative qualification, special-population bridging, arterial–venous concentration characterization, individualized intraoperative “dose-replay” (where actual clinical dosing histories are used as model inputs), and enzyme-contribution verification has not yet been fully reported.

Therefore, the aim of the present study was to develop and comprehensively evaluate a PBPK model for cipepofol across diverse clinical populations. We established the model using the HSK3486-101 healthy-adult dataset and qualified it using the HSK3486-111 healthy-adult dataset and anesthesia induction datasets, extended it to hepatic impairment, renal impairment, elderly, and pediatric populations, and evaluated its predictive performance in surgical patients using individualized dose-replay simulations. The model was further used to characterize arterial–venous concentration behavior and to quantify the relative metabolic contributions of UGT1A9 and CYP2B6, with the goal of providing a mechanistic basis for individualized perioperative dosing.

## 2. Materials and Methods

### 2.1. Clinical Data

Clinical data from nine clinical studies were included in this analysis: NCT03773835 (HSK3486-101), NCT05181007 (HSK3486-111), NCT03808844 (HSK3486-302), NCT04145596 (HSK3486-105), NCT04142970 (HSK3486-106), NCT04197661 (HSK3486-108), NCT04048811 (HSK3486-204), NCT04511728 (HSK3486-306), and ChiCTR2400085640 (HSK3486-404). These datasets covered healthy adults, elective surgery patients, subjects with hepatic impairment, subjects with renal impairment, elderly subjects, and pediatric surgical patients. In total, 371 subjects were included, comprising 1448 arterial and 2073 venous plasma concentration measurements. The corresponding study design, population characteristics, and dosing regimens are summarized in Table S1, and additional details regarding bioanalytical methods, subject eligibility criteria, and data processing procedures are provided in the Supplementary Methods. All studies were approved by the appropriate ethics committees at the participating institutions and conducted in accordance with the Declaration of Helsinki. Written informed consent was obtained from all participants or their legal guardians before enrollment.

### 2.2. Overall Workflow and Software

**Figure 1:**
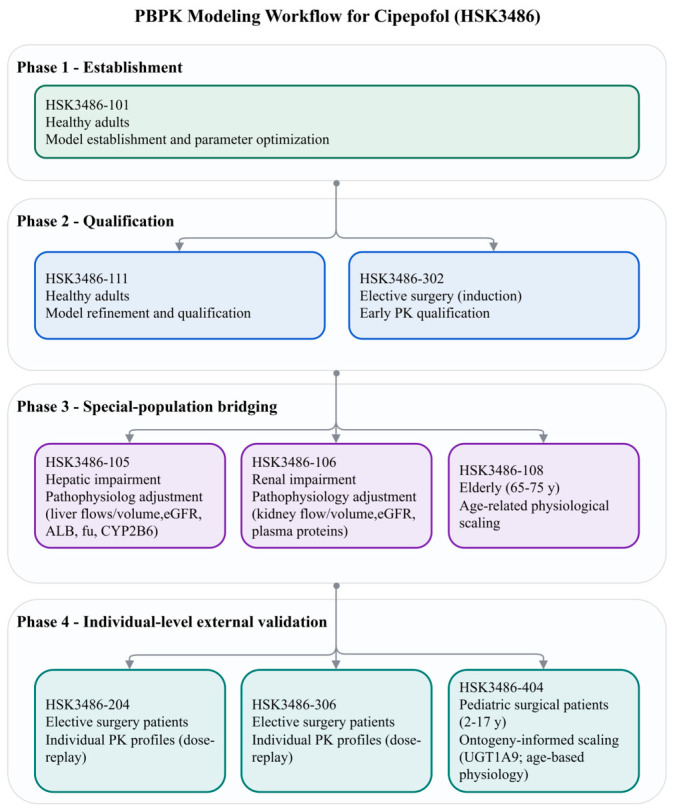
PBPK modeling workflow for cipepofol (HSK3486). The workflow illustrates model establishment in healthy adults using HSK3486-101, followed by model refinement and qualification using the HSK3486-111 healthy-adult dataset and the elective surgery induction study HSK3486-302. The qualified model was subsequently bridged to hepatic impairment (HSK3486-105), renal impairment (HSK3486-106), and elderly subjects (HSK3486-108), and then externally validated at the individual level in adult and pediatric surgical cohorts (HSK3486-204, HSK3486-306, and HSK3486-404).

The overall workflow of the present study is shown in Figure 1. In addition to predicting plasma concentration–time profiles, the model was used to characterize arterial–venous concentration behavior and to estimate the relative contributions of UGT1A9 and CYP2B6 to cipepofol clearance across clinical populations.

PBPK model development and simulation were performed using PK-Sim^®^ (version 11.3) within the Open Systems Pharmacology Suite. Data cleaning and generation of concentration–time profiles and goodness-of-fit (GOF) plots were performed using Python (version 3.13). Exposure metrics, including maximum plasma concentration (C_max_) and area under the plasma concentration–time curve from time zero to the last measurable concentration (AUC_0–t_), were calculated using R (version 4.5.0).

### 2.3. Development of PBPK Model

The physicochemical and biochemical parameters used for model construction are summarized in Table 1. Parameters obtained from literature [15,16] or database sources were fixed during model development, whereas log P_o:w_ and the k_cat_ values for CYP2B6 and UGT1A9 were optimized using the PK-Sim Parameter Identifications module. Details of the parameter identification workflow are provided in the Supplementary Methods.

Cipepofol elimination was implemented primarily through metabolic clearance mediated by UGT1A9 and CYP2B6. As these two pathways together accounted for more than 97% of total metabolic clearance [13], no additional metabolic enzymes or transporters were included in the final model. Because unchanged cipepofol was not detected in urine samples in clinical studies, renal excretion of the parent compound was assumed to be negligible [16]. Accordingly, no additional urinary excretion pathway for unchanged cipepofol was incorporated. However, potential renal metabolic activity was represented by UGT1A9-mediated metabolism in kidney tissue, with tissue-specific UGT1A9 expression defined using the corresponding Expression Profiles settings in the model.

For population-based simulations, a virtual population of 100 East Asian individuals was generated to match the demographic characteristics of the corresponding clinical study as closely as possible. Simulated concentration–time profiles were then compared with the observed data for model evaluation.

### 2.4. Model Extrapolation and External Validation

For subjects with hepatic impairment (HSK3486-105), model extrapolation was performed by adjusting system-specific physiological parameters while retaining the drug-specific parameters established during model development. The adjusted parameters mainly covered organ blood flows, liver volume, estimated glomerular filtration rate (eGFR), hematocrit, plasma protein binding-related factors, and CYP2B6 reference concentration [17,18,19,20,21,22,23,24,25]. The corresponding values for healthy subjects and for mild, moderate, and severe hepatic impairment are summarized in Table S2.

For subjects with renal impairment (HSK3486-106), the main adjustments included organ blood flow, kidney volume, eGFR, hematocrit, and plasma protein scale factor [26,27,28]. The corresponding values for healthy subjects and for mild, moderate, and severe renal impairment are listed in Table S3. Because albumin (ALB) concentrations were required to remain above 35 g/L in this study [8], no additional adjustment was applied to the ALB ontogeny factor.

For elderly subjects (HSK3486-108), anatomical and physiological parameters were automatically scaled in PK-Sim according to age. As this cohort consisted of healthy subjects, no additional disease-related parameter adjustments were introduced. For pediatric surgical patients (HSK3486-404), anatomical and physiological parameters were also automatically scaled in PK-Sim according to age. ALB was assumed to be mature [29], CYP2B6 activity was considered near adult levels by 1 year of age [30], and UGT1A9 ontogeny was implemented according to the PK-Sim ontogeny database and documentation [31].

For the individualized external validation datasets (HSK3486-204, HSK3486-306, and HSK3486-404), simulations were conducted separately for each patient. Individual demographic characteristics and actual dosing information were directly incorporated into the simulations.

### 2.5. Model Evaluation

Model performance was evaluated using graphical approaches across all clinical populations and quantitative approaches where appropriate. Predicted plasma concentration–time profiles were visually compared with the corresponding observed data. When applicable, simulations were performed for both arterial and venous plasma concentrations, and GOF plots were generated by comparing predicted versus observed concentrations on a logarithmic scale, including both population-level and individual-level concentration comparisons.

In the population-level analysis, C_max_ and AUC_0–t_ were derived from the observed and simulated profiles. Specifically, AUC_0–t_ was calculated to the last venous sampling point for venous-only studies, and to the last arterial point for studies with dual sampling. Predictive performance was quantified using fold error (FE) and geometric mean fold error (GMFE). For individualized external validation datasets, conventional exposure metrics such as C_max_ and AUC_0–t_ were not used as evaluation endpoints because the perioperative dosing regimens were highly individualized and often involved repeated bolus administrations and dynamic infusion adjustments. Therefore, model performance in these datasets was assessed primarily by visual comparison of observed and simulated concentration–time profiles for each patient, together with individual-level GOF evaluation. To quantitatively support this individual-level evaluation, point-wise prediction errors were additionally calculated as PE(%)=100×(Pred−Obs)/Obs, with its absolute value denoted as |PE|, and further summarized using the median prediction error (MdPE) and median absolute prediction error (MdAPE). For the population-level exposure evaluation, *FE* and *GMFE* were calculated as follows:

(1) $$FE_{i}=\frac{Pred_{i}}{Obs_{i}}$$

(2) $$GMFE=10^{\frac{1}{n}\sum_{i=1}^{n}\left|\log_{10}\left(FE_{i}\right)\right|}$$

where *Pred*_*i*_ and *Obs*_*i*_ denote the predicted and observed PK values for the *i*-th comparison, respectively. For *FE*, values within two-fold of the observed data were considered acceptable [32]. For *GMFE*, values of 1.0–1.25 were considered excellent, and values ≤2.0 were considered acceptable [33].

A local sensitivity analysis was performed to identify influential parameters affecting arterial C_max_, which was selected as a clinically critical metric since arterial peak concentrations correlate tightly with the maximum depth of anesthesia and provide an essential foundation for future PBPK/PD linkage. Sensitivity coefficients were calculated for key drug-specific, dosing-related, and physiological parameters across representative clinical populations. Parameters were classified as high-impact when the absolute sensitivity coefficient was >0.5, moderate-impact when it was between 0.2 and 0.5, and low-impact when it was <0.2 [34].

To further evaluate the mechanistic plausibility of the model, the fractional contributions of UGT1A9- and CYP2B6-mediated metabolism to total cipepofol clearance were examined as a consistency check against prior experimental evidence. The simulated contribution of each pathway was calculated under the corresponding clinical populations and compared with the previously reported metabolic contribution ratio at 240 h [13,16,35]. This analysis was not intended to independently establish the exact metabolic fractions, but to assess whether the implemented metabolic pathways were consistent with the known metabolism profile of cipepofol.

## 3. Results

### 3.1. Model Development in Healthy Adults, and Anesthesia Induction

**Figure 2:**
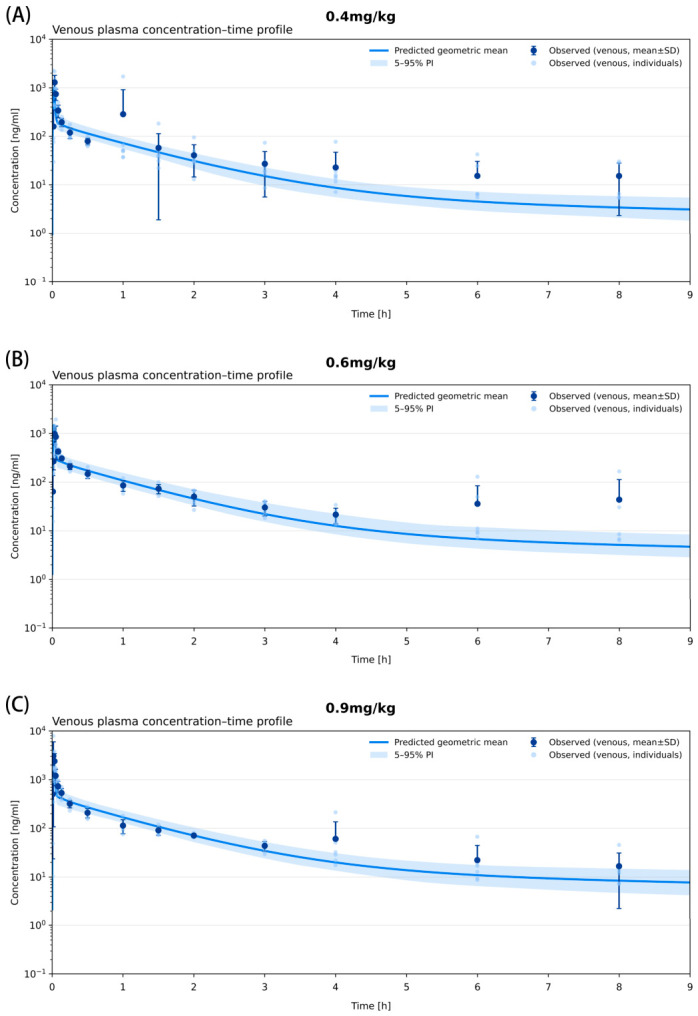
Predicted and observed venous plasma concentration–time profiles of cipepofol in healthy adults after single intravenous administration in HSK3486-101. (**A**) 0.4 mg/kg; (**B**) 0.6 mg/kg; (**C**) 0.9 mg/kg. Solid lines represent the predicted geometric mean concentrations, and shaded areas represent the 5th–95th percentile prediction intervals (5–95% PI). Dark blue symbols with error bars represent observed mean concentrations ± standard deviation (SD), and light blue symbols represent individual observed venous plasma concentrations.

The PBPK model for cipepofol was first established in healthy adults using HSK3486-101. As shown in Figure 2, the model adequately described the venous plasma concentration–time profiles across the three dose levels (0.4, 0.6, and 0.9 mg/kg). The predicted geometric mean profiles were generally consistent with the observed mean data, and most individual observations were contained within the simulated 5th–95th percentile prediction intervals. The model also captured the rapid post-dose decline and the subsequent disposition phase, indicating that the main PK characteristics of cipepofol were reasonably represented in healthy subjects.

**Figure 3:**
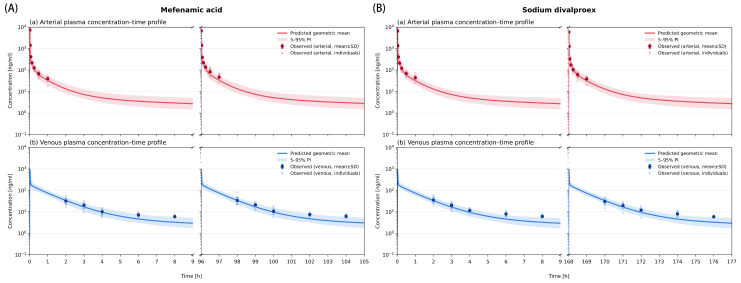
Predicted and observed arterial and venous plasma concentration–time profiles of cipepofol in the HSK3486-111 healthy-adult dataset. (**A**) Mefenamic acid arm; (**B**) Sodium divalproex arm. For each panel, the upper subpanel shows arterial plasma concentrations, and the lower subpanel shows venous plasma concentrations. Solid lines represent the predicted geometric mean concentrations, shaded areas represent the 5–95% PI, dark symbols with error bars represent observed mean concentrations ± SD, and light symbols represent individual observed concentrations. Red indicates arterial plasma and blue indicates venous plasma.

The qualified model was then evaluated using the HSK3486-111 healthy-adult dataset. As shown in Figure 3, the model reasonably reproduced both arterial and venous plasma concentration–time profiles in the presence of mefenamic acid or sodium divalproex. In both populations, arterial concentrations were higher during the early post-dose phase and declined more rapidly than venous concentrations. This supported the ability of the model to characterize arterial–venous concentration differences during the initial distribution phase.

**Figure 4:**
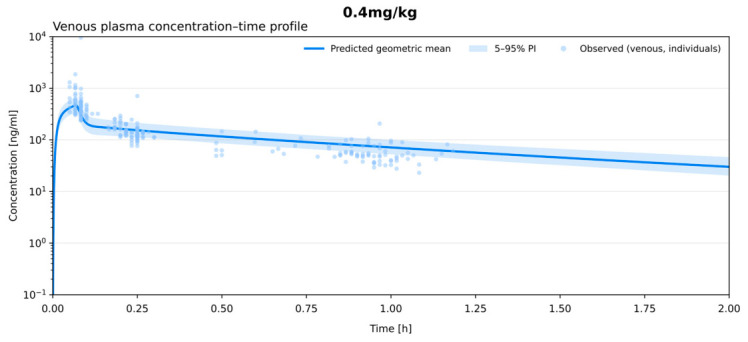
Predicted and observed venous plasma concentration–time profile of cipepofol during anesthetic induction in elective surgery patients in HSK3486-302. The solid line represents the predicted geometric mean concentration, and the shaded area represents the 5–95% PI. Light blue symbols represent individual observed venous plasma concentrations.

Further evaluation in the anesthesia induction study HSK3486-302 showed that the model adequately described the venous concentration–time profile in elective surgery patients (Figure 4). The rapid concentration increase after administration and the subsequent early decline were both captured reasonably well. Collectively, these findings support the adequacy of the model in healthy adults and its robustness across the HSK3486-111 healthy-adult dataset and the anesthesia induction dataset, providing the basis for subsequent extrapolation to special populations and individualized external validation.

### 3.2. Extrapolation of the Qualified Model to Hepatic Impairment, Renal Impairment, and Elderly Subjects

**Figure 5:**
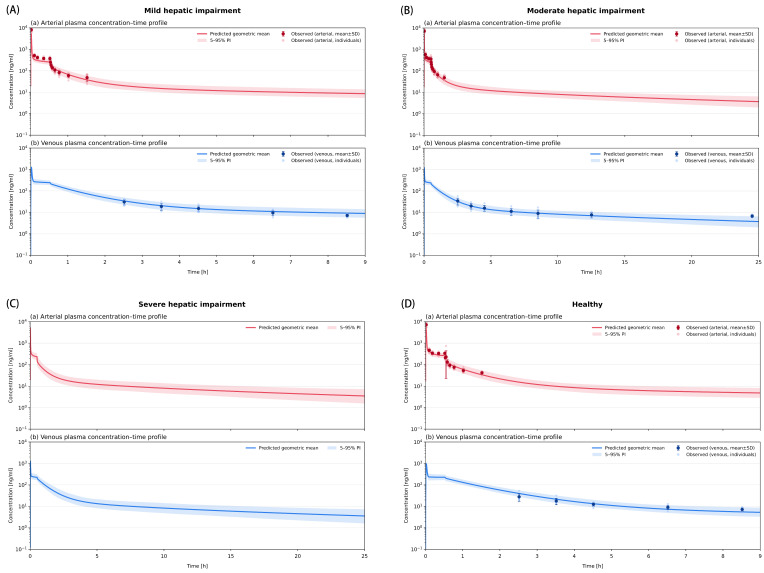
Predicted and observed arterial and venous plasma concentration–time profiles of cipepofol in subjects with hepatic impairment in HSK3486-105. (**A**) Mild hepatic impairment; (**B**) Moderate hepatic impairment; (**C**) Severe hepatic impairment; (**D**) Healthy subjects. Other graphical conventions are the same as those described for Figure 3.

**Figure 6:**
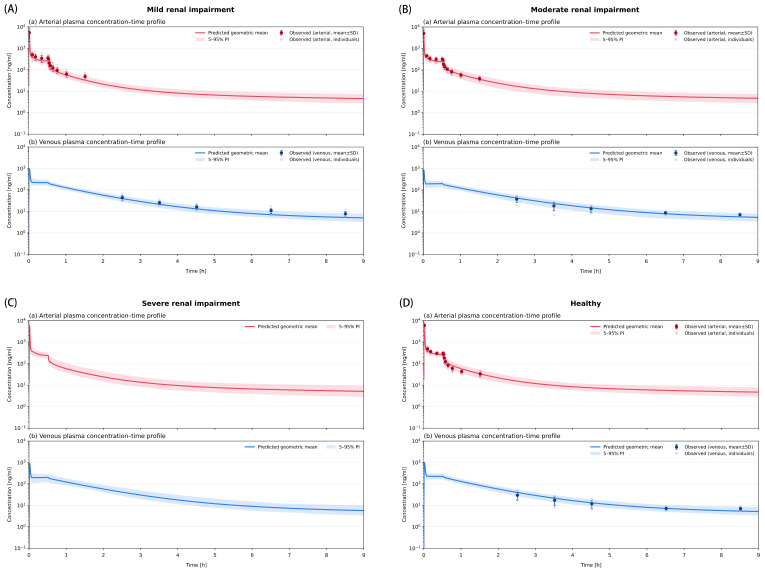
Predicted and observed arterial and venous plasma concentration–time profiles of cipepofol in subjects with renal impairment in HSK3486-106. (**A**) Mild renal impairment; (**B**) Moderate renal impairment; (**C**) Severe renal impairment; (**D**) Healthy subjects. Other graphical conventions are the same as those described for Figure 3.

After qualification in healthy adults and the initial clinical populations, the PBPK model was extrapolated to special populations using pathophysiology-informed physiological adjustments while retaining the optimized drug-specific parameters. In subjects with hepatic impairment (HSK3486-105), the extrapolated model reasonably described both arterial and venous plasma concentration–time profiles across mild, moderate, and severe impairment categories, as well as in healthy controls (Figure 5). Likewise, in subjects with renal impairment (HSK3486-106), the adapted model adequately reproduced the observed arterial and venous concentration–time profiles across renal function groups (Figure 6).

**Figure 7:**
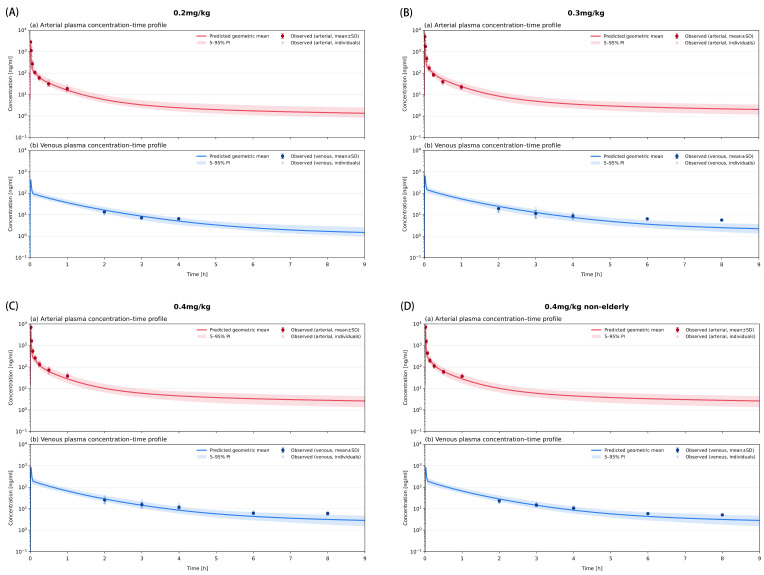
Predicted and observed arterial and venous plasma concentration–time profiles of cipepofol in elderly subjects in HSK3486-108. (**A**) 0.2 mg/kg; (**B**) 0.3 mg/kg; (**C**) 0.4 mg/kg; and (**D**) 0.4 mg/kg in non-elderly subjects. Other graphical conventions are the same as those described for Figure 3.

The model was further evaluated in elderly subjects using HSK3486-108. As shown in Figure 7, the age-scaled model reasonably captured the arterial and venous plasma concentration–time profiles across the evaluated dose levels in elderly subjects and in the non-elderly comparison group. Overall, these findings support the extrapolation capability of the qualified cipepofol PBPK model to hepatic impairment, renal impairment, and elderly populations, and provided the basis for subsequent individualized external validation in adult and pediatric surgical patients.

### 3.3. Individualized External Validation in Adult and Pediatric Surgical Patients

The qualified PBPK model was subsequently applied to individualized external validation in adult and pediatric surgical patients. In adult surgical cohorts from HSK3486-204 and HSK3486-306, individualized simulations based on the actual clinical dosing histories generally reproduced the observed concentration–time profiles under perioperative conditions (Figures S1–S3). Overall agreement at the individual level was acceptable. In pediatric surgical patients from HSK3486-404, the individualized simulations also reasonably captured the observed venous plasma concentration–time profiles across the three age groups (2–5, 6–11, and 12–17 years) (Figures S4–S6). Overall, these findings support the applicability of the qualified cipepofol PBPK model to individualized perioperative PK prediction in both adult and pediatric patients aged 2 to 17 years.

### 3.4. Overall Predictive Performance Based on GOF Plots and Exposure Metrics

**Figure 8:**
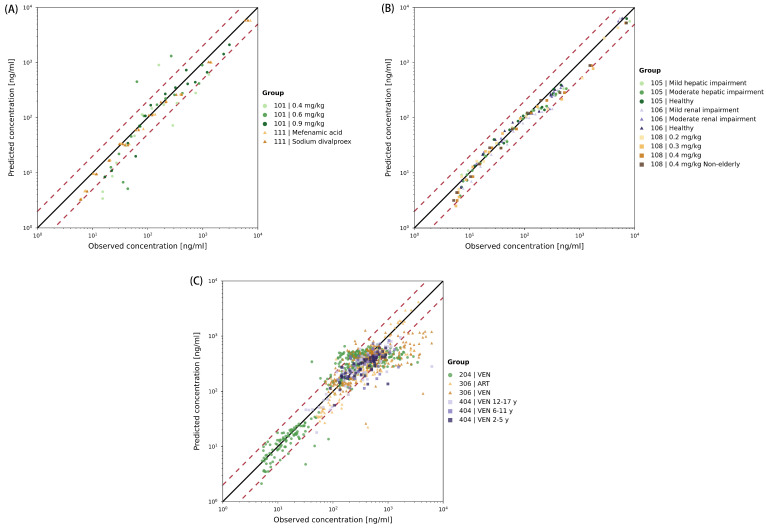
Goodness-of-fit plots comparing PBPK-predicted and observed cipepofol plasma concentrations across model development and validation datasets. (**A**) Healthy-adult datasets (HSK3486-101 and HSK3486-111); (**B**) Special-population bridging studies (HSK3486-105, HSK3486-106, and HSK3486-108); and (**C**) Individual-level external validation studies in adult and pediatric surgical patients (HSK3486-204, HSK3486-306, and HSK3486-404). The solid black line represents the line of identity (observed = predicted), and the dashed red lines represent the two-fold error boundaries. Colors and symbols indicate different studies or subgroups, as defined in the legends.

Overall predictive performance of the qualified cipepofol PBPK model was evaluated using GOF plots and exposure metrics across the model development, special-population bridging, and individualized external validation datasets. As shown in Figure 8, most predicted plasma concentrations were distributed close to the line of identity and remained within the two-fold error boundaries. Specifically, 84.78% (78/92), 96.67% (145/150), and 78.86% (772/979) of data points fell within the two-fold range in Figure 8A, Figure 8B, and Figure 8C, respectively. The highest agreement was observed in the special-population bridging datasets, whereas a broader dispersion was seen in the individualized external validation datasets, consistent with the greater variability expected under real-world perioperative dosing conditions. In addition, sampling-site-specific fold-error performance for arterial and venous plasma concentrations is summarized separately in Table S4, providing a quantitative supplement to the graphical assessment of arterial–venous prediction accuracy. For individualized external validation, the calculated MdPE (and corresponding MdAPE) values were 4.91% (34.87%) for HSK3486-204 venous samples; −32.21% (33.82%) and −15.92% (30.13%) for HSK3486-306 arterial and venous samples; and −20.03% (27.48%) for HSK3486-404 venous samples.

Exposure-based evaluation using C_max_ and AUC_0–t_ further supported the overall predictive performance of the model (Table 2). Across studies, the GMFE ranges were 1.07–1.33 for C_max_ and 1.09–1.27 for AUC_0–t_, with overall GMFE values of 1.22 and 1.21, respectively. Most FE values for both C_max_ and AUC_0–t_ were within the predefined acceptance range of 0.5–2, indicating acceptable agreement between predicted and observed exposure metrics. In addition, as shown in Table S5, the predicted C_max_ and AUC_0–t_ ratios relative to the corresponding healthy or non-elderly comparison groups showed that the exposure differences between hepatic impairment, renal impairment, and elderly groups and their corresponding comparison groups were generally limited and mostly within the commonly used no-effect boundary of 80–125%, suggesting no clinically meaningful PK change from a PK exposure perspective. Taken together, these results support the overall robustness of the cipepofol PBPK model across a broad range of clinical populations.

### 3.5. Sensitivity Analysis of Arterial C_max_

The local sensitivity analysis of arterial C_max_ is summarized in Figure S7. Across clinical populations, arterial C_max_ was mainly influenced by cipepofol lipophilicity, dose, and infusion duration. Lipophilicity showed a consistently high negative influence, whereas dose showed an approximately proportional positive influence and infusion duration showed a negative influence. Plasma protein binding-related parameters, including fraction unbound and plasma protein scale factor, showed moderate effects. In contrast, metabolic enzyme-related parameters had limited influence on arterial C_max_, suggesting that early arterial peak exposure is driven primarily by dosing input, distribution, and binding-related factors rather than metabolic clearance.

### 3.6. Mechanistic Consistency of Simulated UGT1A9- and CYP2B6-Mediated Clearance Contributions

The simulated fractional contributions of UGT1A9- and CYP2B6-mediated metabolism to total cipepofol clearance at 240 h are summarized in Table S6. Across the establishment, qualification, and bridging datasets, cipepofol clearance was generally characterized by an approximate contribution pattern of 2/3 from UGT1A9 and 1/3 from CYP2B6, consistent with previous reports on the major metabolic pathways of cipepofol [13,16,35].

In renal impairment, the relative contribution of CYP2B6 increased with declining renal function, accompanied by a corresponding decrease in the relative contribution of UGT1A9-mediated clearance. This trend became more pronounced in the moderate and severe groups. In severe renal impairment, the simulated fractional contributions were 48.29% for UGT1A9 and 51.62% for CYP2B6, indicating that CYP2B6-mediated metabolism became the predominant pathway.

## 4. Discussion

In this study, we developed and evaluated a clinically oriented PBPK model for cipepofol across multiple clinical populations and perioperative settings. The main contribution of this work is not the development of a new PBPK algorithm, but the establishment of a comprehensive and clinically applicable PBPK framework with a degree of mechanistic interpretability. To our knowledge, this is the first systematic implementation of a cipepofol PBPK model using the open-source PK-Sim platform and the first cipepofol PBPK analysis to include renal impairment and pediatric surgical patients. In addition, this study is the first to perform individualized PBPK dose-replay simulations for cipepofol in real perioperative anesthesia settings using actual intraoperative dosing histories. The model also captured early arterial–venous concentration differences within a unified PBPK framework. Furthermore, the simulated pathway contribution pattern was consistent with the previously reported predominance of UGT1A9 and CYP2B6 as the major metabolic pathways of cipepofol [13]. Collectively, these features extend cipepofol PBPK modeling from conventional exposure prediction toward a clinically relevant framework for special-population extrapolation and individualized perioperative simulation.

The present study advances the clinical PK understanding of cipepofol by developing a comprehensive PBPK framework validated across diverse clinical populations. Previous population PK/PD analysis was valuable for identifying major covariates such as body weight, age, and sampling site, and for quantifying their influence on perioperative exposure-response relationships [14]. In addition, a population PK-based exposure-safety analysis characterized cipepofol pharmacokinetics and evaluated the association between exposure and hypotension in mechanically ventilated patients [36]. Likewise, the previously reported PBPK work established an important basis for physiological-based dose projections and model-informed decision-making in specific populations [15]. Building on these prior efforts, the current study demonstrates the robust interpretability of a mechanistic framework across the diverse clinical populations of intravenous anesthesia. Notably, cipepofol disposition is governed not only by patient-specific covariates but also by the combined effects of rapid tissue distribution, early-phase arterial–venous concentration gradients, and the intricate dosing regimens of perioperative care. In this context, the value of the present model lies in its ability to integrate these physiological, dosing, and sampling complexities, thereby supporting individualized dose-replay simulations and extrapolation in various special populations as well as real-world surgical settings.

A notable strength of the present model is its ability to describe early arterial–venous concentration disequilibrium. This feature is pharmacologically important for rapidly acting intravenous anesthetics, because early plasma concentrations after intravenous administration are determined not only by systemic distributional equilibrium, but also by incomplete vascular mixing, cardiac output-dependent redistribution, rapid tissue distribution, and pulmonary handling. Similar arterial–venous differences have long been reported for propofol, particularly during the first minutes after dosing, and pulmonary uptake has also been shown to contribute to its early disposition [37,38,39]. In the current study, the model reproduced the observed pattern that arterial concentrations were higher in the early phase and declined more rapidly than venous concentrations, including after dosing interruption. These findings indicate that the blood sampling site should be considered explicitly when interpreting early cipepofol PK data, especially in anesthesia induction, and they further support the physiological credibility of the model. Nevertheless, the present analysis did not provide a quantitative mechanistic breakdown of the individual contributions of lung uptake, cardiac output, tissue partitioning, or other physiological drivers to the arterial–venous differences. Therefore, the arterial–venous interpretation should be regarded as a physiologically plausible explanation based on the observed clinical pattern and prior evidence from related intravenous anesthetics, rather than a formal quantitative decomposition of the underlying mechanisms.

The hepatic impairment study of cipepofol showed that AUC increased slightly with worsening liver function, whereas PD measures changed little, supporting no dose adjustment for subjects with mild or moderate hepatic impairment [7]. A similar pattern was reproduced by our PBPK model when simulations were extended beyond the early perioperative period. In contrast, the differences became much smaller when the analysis was confined to the perioperative period and early arterial exposure. This discrepancy is pharmacologically plausible for a highly protein-bound, rapidly distributing intravenous anesthetic. Although hepatic dysfunction may reduce metabolic clearance and thereby increase later overall exposure, early arterial concentrations after dosing are influenced more strongly by rapid mixing, tissue distribution, and arterial–venous disequilibrium than by elimination alone. In addition, impairment-related changes in plasma protein binding and distribution may partly offset the expected increase in total plasma concentrations during the early phase. Therefore, exposure evaluated over a longer post-dose interval mainly reflects overall systemic disposition, whereas early arterial exposure within the perioperative dosing window may be more relevant for perioperative interpretation.

The renal impairment study showed only modest changes in cipepofol exposure and no meaningful PD differences, suggesting that dose adjustment is not required for mild-to-moderate renal impairment in patients with preserved ALB (>35 g/L) [8]. Our simulations were generally consistent with this conclusion, but renal impairment deserves additional mechanistic consideration. In the present model, cipepofol clearance was assumed to be mediated mainly by UGT1A9 and CYP2B6, with approximate contributions of 2/3 and 1/3, respectively. Unlike CYP2B6, which is primarily hepatic, UGT1A9 is expressed in both liver and kidney [40,41]. Consequently, as renal impairment worsens, the model predicted a progressive decline in the relative contribution of UGT1A9-mediated clearance, which was accompanied by a reciprocal increase in the relative dominance of hepatic CYP2B6 metabolism. This predicted pathway redistribution is biologically plausible, as it aligns with the expected reduction in kidney-associated metabolic capacity. Nevertheless, this finding remains model-based, as direct quantitative data on UGT1A9 expression, activity, or pathway-specific contribution in renal impairment were not available. Moreover, no dedicated mechanistic sensitivity analysis was performed to isolate the individual physiological drivers of this predicted shift. Therefore, the pathway contribution analysis should be interpreted as a model-based consistency assessment with biological plausibility, rather than causal confirmation of pathway redistribution. This predicted pathway shift may also have implications for drug–drug interaction (DDI) risk assessment. If the relative contribution of CYP2B6 becomes more prominent in advanced renal impairment, cipepofol exposure may theoretically become more susceptible to CYP2B6 inhibition or induction in this population. However, because dedicated clinical DDI data in renal impairment were not available, this implication should be considered hypothesis-generating rather than definitive.

In elderly subjects, the age-dependent scaling implemented in PK-Sim was sufficient to provide acceptable predictions without additional disease-specific adjustment. However, because this dataset mainly included healthy older adults younger than 75 years, extrapolation to very old, frail, or multimorbid patients should remain cautious. The simulated exposure differences in hepatic/renal impairment and elderly subjects were modest, indicating a low risk of exposure-driven excessive anesthesia or delayed recovery. However, clinical response depends on effect-site equilibration, patient vulnerability, and perioperative titration. Thus, the conclusion of “no clinically meaningful change” is strictly limited to PK exposure rather than PD response.

In pediatric patients, the model also showed reasonable performance across the 2–17-year age range. This supports the feasibility of age-appropriate extrapolation when physiological growth and enzyme ontogeny are incorporated through a mechanism-based approach, as described in the Methods. However, children younger than 2 years were not investigated in the present study, and no clinical PK data were available to validate model performance in this age group. Therefore, the pediatric extrapolation should be interpreted as applicable to children aged 2 years and older, while extrapolation to younger children requires additional caution. Uncertainty in UGT1A9 and CYP2B6 ontogeny should also be considered when interpreting the pediatric extrapolation. Although established PK-Sim ontogeny functions were used, developmental enzyme expression may vary across individuals and may be less certain in younger children, in whom hepatic and extrahepatic metabolic capacity may still be maturing. Therefore, the current pediatric predictions should be interpreted in the context of the available 2–17-year clinical validation data, and additional PK data or sensitivity analyses of enzyme ontogeny assumptions would be useful for further extrapolation to younger children.

Another strength of this study is the individualized external validation using actual clinical dosing records in both adult and pediatric surgical patients. Such dose-replay simulations provide a more stringent test of model performance under real perioperative conditions than validation that relies solely on fixed dosing regimens or average population profiles [42,43]. Although these individualized simulations incorporated real-world dosing histories, potentially relevant perioperative factors, such as hemodynamic fluctuations, fluid shifts, concomitant medications, and surgery-related variability, were not explicitly accounted for. Therefore, the greater variability observed relative to the development and bridging datasets was expected. Nevertheless, the overall agreement supports the practical utility of the model for perioperative PK prediction and provides a basis for future integration with model-informed individualized dosing and mechanistic model extension.

Future studies incorporating more intensive early arterial and venous sampling, targeted pulmonary uptake data, and dedicated mechanistic sensitivity analyses are needed to quantify the relative contributions of key physiological drivers to early arterial–venous disequilibrium after cipepofol administration. Integration of PBPK simulations with PD endpoints, such as anesthesia depth, recovery profiles, and hemodynamic outcomes, may help clarify the clinical relevance of the predicted exposure differences. In addition, the model-predicted alteration in metabolic pathway contributions under renal impairment warrants further clinical or translational evaluation using metabolite profiling, enzyme activity data, or renal impairment-specific physiological measurements.

## 5. Conclusions

In conclusion, we developed and comprehensively evaluated a PBPK model for cipepofol across healthy adults, anesthesia induction, special populations, and individualized perioperative settings. The model adequately described plasma concentration–time profiles, captured early arterial–venous concentration differences, and showed acceptable predictive performance at both the population and individual levels. Extrapolation to hepatic impairment, renal impairment, elderly subjects, and pediatric patients was generally acceptable, with no clinically meaningful PK changes observed in the evaluated adult special populations; however, these findings should be interpreted in the context of the available clinical datasets and model assumptions. Overall, this work provides a mechanistic framework for characterizing cipepofol disposition in clinically relevant anesthesia populations and may serve as a hypothesis-generating tool for future model-informed dosing studies and prospective evaluation in perioperative care.

## Figures and Tables

**Table 1:** Key physicochemical and biochemical parameters used in the PBPK model of cipepofol.

Parameters	Unit	Value	Reference
Molecular weight	g/mol	204.31	Drug Bank
log P_o:w_	/	3.01	Optimized ^a^
f_u, plasma_	/	0.051	(15)
pKa (Acid)	/	10.95	Drug Bank
Solubility	μg/mL	9.00	Drug Bank
Partition coefficients	/	PK-Sim Standard	/
Cellular permeabilities	/	PK-Sim Standard	/
CYP2B6 K_m_	μmol/L	4.74	(16)
CYP2B6 k_cat_	1/min	18.54	Optimized ^b^
UGT1A9 K_m_	μmol/L	1.96	(16)
UGT1A9 k_cat_	1/min	116.24	Optimized ^b^

log P_o:w_—log octanol-water partition coefficient; f_u,plasma_—fraction unbound in plasma; pKa—acid dissociation constant; K_m_—Michaelis–Menten constant; k_cat_—catalytic rate constant, number of substrate molecules converted per enzyme site per unit time at maximum efficiency. ^a^ In PK-Sim, log P was used as a surrogate for membrane affinity (log MA); a reasonable variation around the log P value was allowed during optimization using the Parameter Identifications module. ^b^ Optimized using the PK-Sim Parameter Identifications module, with Monte-Carlo followed by Levenberg–Marquardt optimization.

**Table 2:** Exposure-based evaluation of PBPK model predictive performance (C_max_ and AUC_0–t_) across clinical studies.

Study No.	Group	C_max_ [ng/mL]			AUC_0–t_ [h*ng/mL]		
		Prediction	Observation	FE	Prediction	Observation	FE
HSK3486-101	0.4 mg/kg	1054.65	1291.00	0.82	282.02	433.18	0.65
	0.6 mg/kg	1444.56	978.67	1.48	389.63	473.60	0.82
	0.9 mg/kg	2333.05	3049.60	0.77	618.94	673.87	0.92
	GMFE			1.33			1.27
HSK3486-111	Mefenamic acid	6580.08	7372.50	0.89	217.47	196.12	1.11
	Sodium divalproex	6522.37	6584.44	0.99	214.77	187.57	1.15
	GMFE			1.07			1.13
HSK3486-105	Mild hepatic impairment	5758.05	8105.00	0.71	450.16	603.44	0.75
	Moderate hepatic impairment	5314.37	7141.25	0.74	466.40	573.61	0.81
	Severe hepatic impairment	5172.64	/	/	485.62	/	/
	Healthy	6727.55	7170.00	0.94	434.08	534.39	0.81
	GMFE			1.26			1.27
HSK3486-106	Mild renal impairment	6464.88	5386.12	1.20	451.30	487.76	0.93
	Moderate renal impairment	5776.01	5041.25	1.15	493.02	443.34	1.11
	Severe renal impairment	5766.05	/	/	522.07	/	/
	Healthy	6501.97	5941.25	1.09	431.37	468.65	0.92
	GMFE			1.15			1.09
HSK3486-108	0.2 mg/kg	3144.47	2802.50	1.12	138.67	101.57	1.37
	0.3 mg/kg	4667.31	4975.00	0.94	164.17	160.70	1.02
	0.4 mg/kg	5924.91	6880.00	0.86	211.51	208.03	1.02
	0.4 mg/kg Non-elderly	6531.87	7056.25	0.93	220.33	189.50	1.16
	GMFE			1.10			1.13
GMFE				1.22			1.21

C_max_—maximum plasma concentration; AUC_0–t_—area under the plasma concentration–time curve from time zero to the last measurable concentration; FE—fold error; GMFE—geometric mean fold error.

## Data Availability

The data supporting this study are not publicly available due to privacy and ethical considerations.

## Supplementary Materials

The following supporting information can be downloaded at: https://www.mdpi.com/article/10.3390/pharmaceutics18060763/s1, Supplementary Methods; Table S1. Summary of clinical data for Cipepofol; Table S2. Parameter adaptations for hepatic impairment populations in the cipepofol PBPK model; Table S3. Parameter adaptations for renal impairment populations in the cipepofol PBPK model; Table S4. Sampling-site-specific prediction accuracy for arterial and venous plasma concentrations based on the 0.5–2-fold criterion; Table S5. Predicted exposure ratios of C_max_ and AUC_0–t_ across special-population groups relative to corresponding control groups; Table S6. Simulated UGT1A9- and CYP2B6-mediated clearance contributions at 240 h and their consistency with prior evidence across different clinical populations; Figure S1. Predicted and observed venous plasma concentration–time profiles of cipepofol in individual patients from HSK3486-204; Figure S2. Predicted and observed arterial plasma concentration–time profiles of cipepofol in individual patients from HSK3486-306; Figure S3. Predicted and observed venous plasma concentration–time profiles of cipepofol in individual patients from HSK3486-306; Figure S4. Predicted and observed venous plasma concentration–time profiles of cipepofol in individual pediatric patients aged 2–5 years from HSK3486-404; Figure S5. Predicted and observed venous plasma concentration–time profiles of cipepofol in individual pediatric patients aged 6–11 years from HSK3486-404; Figure S6. Predicted and observed venous plasma concentration–time profiles of cipepofol in individual pediatric patients aged 12–17 years from HSK3486-404; Figure S7. Local sensitivity analysis of arterial C_max_ across clinical populations and dosing scenarios; Supplementary References.

## Abbreviations

The following abbreviations are used in this manuscript:

ALB	Albumin
AUC_0–t_	Area under the plasma concentration–time curve from time zero to the last measurable concentration
C_max_	Maximum plasma concentration
CYP2B6	Cytochrome P450 2B6
DDI	Drug–drug interaction
eGFR	Estimated glomerular filtration rate
FE	Fold error
GMFE	Geometric mean fold error
GOF	Goodness-of-fit
MdAPE	Median absolute prediction error
MdPE	Median prediction error
PBPK	Physiologically based pharmacokinetic
PK	Pharmacokinetics
PK/PD	Pharmacokinetics/pharmacodynamics
UGT	UDP-glucuronosyltransferase
