# Supplementary material for: Development and Evaluation of a Physiologically Based Pharmacokinetic Model for Cipepofol Across Diverse Clinical Populations
Source: Pharmaceutics. 2026 Jun 22;18(6):763. doi: 10.3390/pharmaceutics18060763 (PMC13306506; doi:10.3390/pharmaceutics18060763)
Supplement: Supplementary file 1 [file pharmaceutics-18-00763-s001.zip › pharmaceutics-4358655-supplementary.pdf]

Supplementary Materials

Development and Evaluation of a Physiologically Based Pharmacokinetic Model for Cipepofol Across Diverse Clinical Populations

Junmin Li [1], Longjie Li [2], Fangbin Ding [2], Meixia Chen [3], Mengyue Hu [3], Xiaoqiang Xiang [2,4,5,*] and Jing Tang [1,*]

[1] Obstetrics & Gynecology Hospital of Fudan University, Shanghai Key Lab of Reproduction and Development, Shanghai Key Lab of Female Reproductive Endocrine Related Diseases, 200433, Shanghai, China

[2] Department of Clinical Pharmacy and Pharmacy Administration, School of Pharmaceutical Sciences, Fudan University, 201203, Shanghai, China

[3] Haisco Pharmaceutical Group Co. Ltd., 856000, Chengdu, China

[4] Quzhou Fudan Institute, 324003, Quzhou, China

[5] State Key Laboratory of Advanced Drug Formulations for Overcoming Delivery Barriers, 201203, Shanghai, China

Correspondence: xiangxq@fudan.edu.cn (X.X.); tjfc2020@163.com (J.T.)

Contents

Supplementary Methods 3

Table S1 Summary of clinical data for Cipepofol 5

Table S2 Parameter adaptations for hepatic impairment populations in the cipepofol PBPK model 7

Table S3 Parameter adaptations for renal impairment populations in the cipepofol PBPK model 8

Table S4 Sampling-site-specific prediction accuracy for arterial and venous plasma concentrations based on the 0.5-2-fold criterion 9

Table S5 Predicted exposure ratios of C_{max} and AUC_{0-t} across special-population groups relative to corresponding control groups 10

Table S6 Simulated UGT1A9- and CYP2B6-mediated clearance contributions at 240 h and their consistency with prior evidence across different clinical populations. 11

Figure S1 Predicted and observed venous plasma concentration-time profiles of cipepofol in individual patients from HSK3486-204 12

Figure S2 Predicted and observed arterial plasma concentration-time profiles of cipepofol in individual patients from HSK3486-306 15

Figure S3 Predicted and observed venous plasma concentration-time profiles of cipepofol in individual patients from HSK3486-306 17

Figure S4 Predicted and observed venous plasma concentration-time profiles of cipepofol in individual pediatric patients aged 2-5 years from HSK3486-404 22

Figure S5 Predicted and observed venous plasma concentration-time profiles of cipepofol in individual pediatric patients aged 6-11 years from HSK3486-404....24

Figure S6 Predicted and observed venous plasma concentration-time profiles of cipepofol in individual pediatric patients aged 12-17 years from HSK3486-404 .25

Figure S7 Local sensitivity analysis of arterial C_{max} across clinical populations and dosing scenarios 26

Supplementary References 27

Supplementary Methods

Clinical data sources, subject eligibility, and quality control

Plasma cipepofol concentrations were quantified using a validated liquid chromatography-tandem mass spectrometry (LC-MS/MS) method. Blood samples (3 mL) were collected before dosing and at protocol-defined time points after administration. Following collection, samples were centrifuged at 4°C at 1700×*g* for 10 min to obtain plasma, and plasma samples were stored at -80°C until analysis. The lower limit of quantification was 5 ng/mL.

Subjects were eligible for inclusion in the PBPK analysis if they had adequately documented cipepofol exposure and sufficient pharmacokinetic observations. Specifically, subjects were required to have at least one recorded cipepofol administration, explicitly documented blood sampling sites (arterial or venous), and at least three evaluable plasma concentration measurements. Subjects were excluded if sampling times could not be reliably verified or if key covariates required for simulation were missing, including age, body weight, or sex. For the hepatic impairment study, albumin (ALB) information was additionally required for subject inclusion, whereas both ALB and estimated glomerular filtration rate (eGFR) were required for the renal impairment study.

Prior to model analysis, concentration–time records were reviewed for data quality. Concentrations below the lower limit of quantification were excluded from analysis. Records containing obvious data entry errors were removed after manual review, and missing concentration values were not imputed. After data cleaning, the resulting dataset was used for PBPK model establishment, qualification, special-population bridging, and external validation.

Parameter optimization procedure

Parameter optimization was performed using the Parameter Identifications module in PK-Sim following the workflow and optimization procedures recommended in the PK-Sim documentation. Only three drug-specific parameters were optimized during model development, namely log $P_{o:w}$, CYP2B6 k_{cat}, and UGT1A9 k_{cat}, whereas the remaining physicochemical, biochemical, and system-specific parameters were fixed

according to literature sources, database values, or PK-Sim standard methods.

A two-step optimization strategy was applied. First, a Monte-Carlo algorithm was used for global parameter search. For this step, observed and simulated values below the lower limit of quantification (LLOQ) were set to the LLOQ, and no observed data below the LLOQ were removed. The break condition for relative error improvement was set to 0.001, the scale of projection degree (alpha) was set to 30, and the maximum number of iterations was set to 10,000. Second, the parameter estimates obtained from the Monte-Carlo search were further refined using the Levenberg-Marquardt algorithm. For the Levenberg-Marquardt step, the relative chi-square convergence criterion (ftol) was set to 0.001, the relative parameter convergence criterion (xtol) was set to 1E-06, the orthogonality convergence criterion (gtol) was set to 1E-10, the initial step bound factor was set to 100, the maximum number of iterations was set to 200, and the finite derivative step size was set to 1E-09.

The final parameter set was selected based on objective-function reduction, visual agreement between predicted and observed concentration-time profiles, biological plausibility of the optimized values, and predictive performance in subsequent model qualification and validation datasets. Correspondingly, formal parameter confidence intervals and parameter-correlation matrices were not generated, reflecting the methodological characteristic that the deterministic PK-Sim parameter-identification workflow does not provide a likelihood-based covariance framework comparable to nonlinear mixed-effects modeling. Consequently, the reliability and identifiability of the optimized values were evaluated pragmatically through robust validation across independent datasets rather than formal statistical uncertainty formalisms.

Table S1 Summary of clinical data for Cipepofol.

Study No.	Phase	N	Population (Chinese)	Dose Regimen	PK Sampling Schedule
**NCT03773835 (HSK3486-101)**	I	7	Healthy	0.4 mg/kg, IV bolus	Venous: 0.5, 1, 2, 3, 5, 8, 15, 30 min, 1, 1.5, 2, 3, 4, 6, 8, 24 h post-infusion.
		6	Healthy	0.6 mg/kg, IV bolus	
		6	Healthy	0.9 mg/kg, IV bolus	
**NCT05181007 (HSK3486-111)**	I	20	Healthy (mefenamic acid arm)	Cipepofol: 0.4 mg/kg, IV infusion 1 min<br>- Day 1 (alone)<br>- Day 5, 2h after the 5th dose of mefenamic acid<br>Mefenamic acid: 500 mg PO loading dose, followed by 250 mg PO q6h × 8 doses<br>- Day 4–Day 5	Arterial: 1, 2, 4, 8, 15, 30 min and 1 h after administration.<br>Venous: 2, 3, 4, 6, 8 h after administration.
		18	Healthy (sodium divalproex arm)	Cipepofol: 0.4 mg/kg, IV infusion 1 min<br>- Day 1 (alone)<br>- Day 8, 2 h after sodium divalproex<br>Sodium divalproex: 1000 mg PO q.d. × 5 doses<br>- Day 4–Day 8	Arterial: 1, 2, 4, 8, 15, 30 min and 1 h after administration.<br>Venous: 2, 3, 4, 6, 8 h after administration.
**NCT03808844 (HSK3486-302)**	III	80	Elective surgery patients	0.4 mg/kg, IV bolus	Venous: 5, 15, 60 min post-infusion.
**NCT04145596 (HSK3486-105)**	I	8	Mild hepatic impairment	0.4 mg/kg IV infusion 1 min → 0.4 mg/kg/h for 30 min (maintenance)	Arterial: end of induction infusion (1 min); 5, 10, 20, 30 min after start of maintenance infusion; 1, 2, 4, 8, 15, 30 min and 1 h after the end of the maintenance infusion.<br>Venous: 2, 3, 4, 6, 8, 12, 24 h after the end of the maintenance infusion.
		8	Moderate hepatic impairment		
		8	Healthy		
**NCT04142970 (HSK3486-106)**	I	8	Mild renal impairment	0.4 mg/kg IV infusion 1 min → 0.4 mg/kg/h for 30 min (maintenance)	Arterial: end of induction infusion (1 min); 5, 10, 20, 30 min after start of maintenance infusion; 1, 2, 4, 8, 15, 30
		8	Moderate renal impairment		

		8	Healthy		min and 1 h after the end of the maintenance infusion.<br>Venous: 2, 3, 4, 6, 8 h after the end of the maintenance infusion.
**NCT04197661**<br>**(HSK3486-108)**	I	8	65-75 y, elderly	0.2 mg/kg, IV bolus	Arterial: 1, 2, 4, 8, 15, 30 min and 1 h after the start of infusion.<br>Venous: 2, 3, 4, 6, 8 h post-dose.
		8	65-75 y, elderly	0.3 mg/kg, IV bolus	
		8	65-75 y, elderly	0.4 mg/kg, IV bolus	
		8	Non-elderly	0.4 mg/kg, IV bolus	
**NCT04048811**<br>**(HSK3486-204)**	II	30	Elective surgery patients	Induction: 0.4 mg/kg, IV bolus<br>Maintenance: 1.0 ± 0.5 mg/kg/h, IV infusion (adjustable)	Venous: start of intubation; 1, 2, 5, 10 min after the initial maintenance administration; within 2 min after each dose adjustment; 15min, 1, 6, 10, 24, 48 h after the end of the maintenance infusion.
**NCT04511728**<br>**(HSK3486-306)**	III	86	Elective surgery patients	Induction: 0.4 mg/kg, IV bolus<br>Maintenance: 0.8 mg/kg/h, IV infusion (adjustable in increments of 0.1–0.4 mg/kg/h; max 2.4 mg/kg/h)	Arterial/Venous: end of induction infusion; 15-45 min, 1-1.5 h after the start of maintenance infusion; 0 and 1-6 h after the end of the maintenance infusion.
**ChiCTR 2400085640**<br>**(HSK3486-404)**	IV	12	12-17 y, elective surgery patients	Induction: 0.5 mg/kg, IV bolus (up to 2 top-up doses, each 50% of the initial dose)<br>Maintenance: 1.0 mg/kg/h, IV infusion (adjustable; 0.5–3.0 mg/kg/h)	Immediately after anesthesia induction; 10-15 min after start of maintenance; within 10 min, 30-60 min, and 120-360 min* after end of administration.<br>*120–360 min point is for ages 12–17 years only
		12	6-11 y, elective surgery patients	Induction: 0.6 mg/kg, IV bolus (up to 2 top-up doses, each 50% of the initial dose)<br>Maintenance: 1.2 mg/kg/h (later increased to 1.4 mg/kg/h), IV infusion (adjustable; 0.6–4.0 mg/kg/h)	
		14	2-5 y, elective surgery patients	Same dosing regimen as Age 6–11 years	

IV - intravenous; PO - oral.

Table S2 Parameter adaptations for hepatic impairment populations in the cipepofol PBPK model.

Parameter	Healthy	Mild	Moderate	Severe	Ref
Blood flow rate (L/min)					
Liver	0.39	0.44	0.55	0.51	(17)
Kidney	1.04	0.91	0.70	0.51	(18)
Portal	0.94	0.37	0.35	0.04	(17)
Other organs (fractions of healthy)	1	1.75	2.25	2.75	(19)
Liver volume (L)	1.91	1.32	1.05	0.53	(19)
eGFR (mL/min/1.73 m^2)	93.37	90.03	69.45	35.16	(20-22)
Hematocrit	0.47	0.39	0.37	0.35	(23)
Ontogeny factor (albumin)	1.00	1.07	0.85	0.50	Study dataset
Ontogeny factor (α1--acid glycoprotein)	1.00	0.60	0.56	0.30	(24)
$f_{u,\ plasma}$	0.05	0.05	0.06	0.10	(19)
CYP2B6 reference concentration (μmol/L)	1.56	1.56	1.40	1.25	(25)

eGFR - estimated glomerular filtration rate; $f_{u,\ plasma}$ - fraction unbound in plasma.

Table S3 Parameter adaptations for renal impairment populations in the cipepofol PBPK model.

Parameter	Healthy	Mild	Moderate	Severe	Ref
Blood flow rate (L/min)					
Liver	0.38	0.38	0.38	0.34	(26)
Kidney	1.00	1.02	0.37	0.25	(27)
Kidney volume (L)	0.34	0.34	0.25	0.21	(27)
eGFR (mL/min/1.73 m^2)	128.60	72.70	43.00	25.00	Study dataset
Hematocrit	0.47	0.47	0.45	0.43	(28)
Plasma protein scale factor	1.00	1.00	1.07	1.16	(28)
Ontogeny factor (albumin)	1.00	1.00	1.00	1.00	Study dataset

eGFR - estimated glomerular filtration rate.

Table S4 Sampling-site-specific prediction accuracy for arterial and venous plasma concentrations based on the 0.5-2-fold criterion.

Study No.	Sampling site	Total observations	Observations within 0.5-2-fold range	Percentage (%)
HSK3486-101	venous	44	30	68.18
HSK3486-111	arterial	28	28	100.00
	venous	20	20	100.00
HSK3486-105	arterial	36	36	100.00
	venous	17	17	100.00
HSK3486-106	arterial	36	35	97.22
	venous	15	15	100.00
HSK3486-108	arterial	28	25	89.29
	venous	18	17	94.44
HSK3486-204	venous	408	317	77.70
HSK3486-306	arterial	116	93	80.17
	venous	291	223	76.63
HSK3486-404	venous	164	139	84.76
Total	arterial	244	217	88.93
	venous	977	778	79.63

Table S5 Predicted exposure ratios of C_{max} and $AUC_{0\text{-}t}$ across special-population groups relative to corresponding control groups.

**Population**	**Group**	**C_{max} ratio vs control**	**$AUC_{0\text{-}t}$ ratio vs control**
Hepatic impairment	Mild	85.59%	103.70%
	Moderate	78.99%	107.45%
	Severe	76.89%	111.88%
Renal impairment	Mild	99.43%	104.62%
	Moderate	88.83%	114.29%
	Severe	88.68%	121.02%
Elderly	0.4 mg/kg elderly vs non-elderly	90.71%	96.00%

Table S6 Simulated UGT1A9- and CYP2B6-mediated clearance contributions at 240 h and their consistency with prior evidence across different clinical populations.

Study No.	Group	UGT1A9 (%)	CYP2B6 (%)
**HSK3486-101**	0.4 mg/kg	62.67	37.30
	0.6 mg/kg	62.75	37.24
	0.9 mg/kg	62.65	37.33
**HSK3486-111**	Mefenamic acid arm	61.95	38.02
	Sodium divalproex arm	62.34	37.64
**HSK3486-302**	0.4 mg/kg	62.92	37.03
**HSK3486-105**	Mild hepatic impairment	63.58	36.39
	Moderate hepatic impairment	62.34	37.62
	Severe hepatic impairment	60.37	39.58
	Healthy	61.77	38.13
**HSK3486-106**	Mild renal impairment	61.67	38.20
	Moderate renal impairment	52.07	47.79
	Severe renal impairment	48.29	51.62
	Healthy	61.66	38.26
**HSK3486-108**	0.2 mg/kg	62.41	37.48
	0.3 mg/kg	62.43	37.46
	0.4 mg/kg	62.40	37.51
	0.4 mg/kg Non-elderly	61.85	38.09

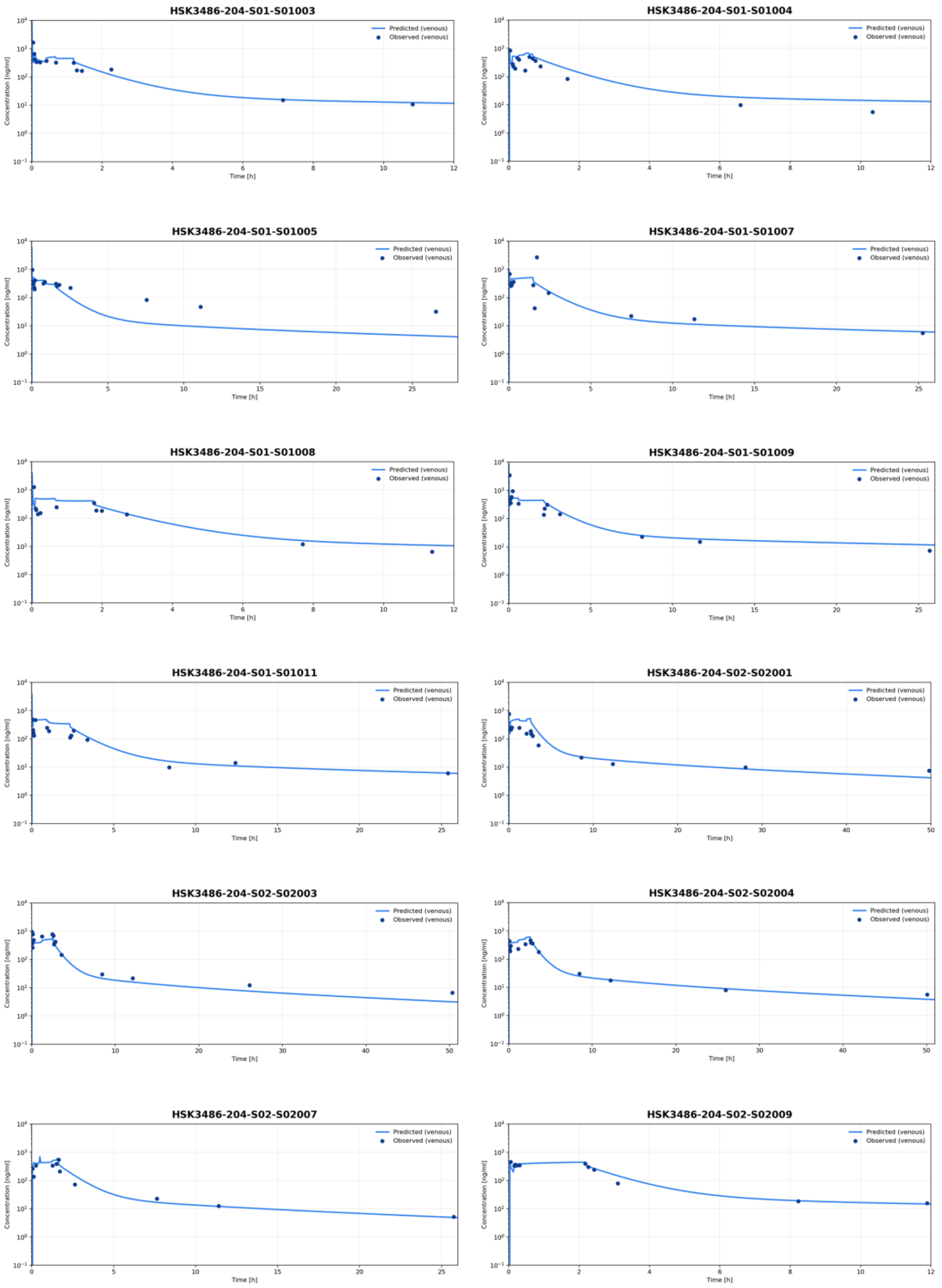

Figure S1 Predicted and observed venous plasma concentration-time profiles of cipepofol in individual patients from HSK3486-204.

Each subpanel represents one patient. The solid blue lines represent model-predicted venous plasma concentrations, and the dark blue symbols represent the corresponding observed venous plasma concentrations.

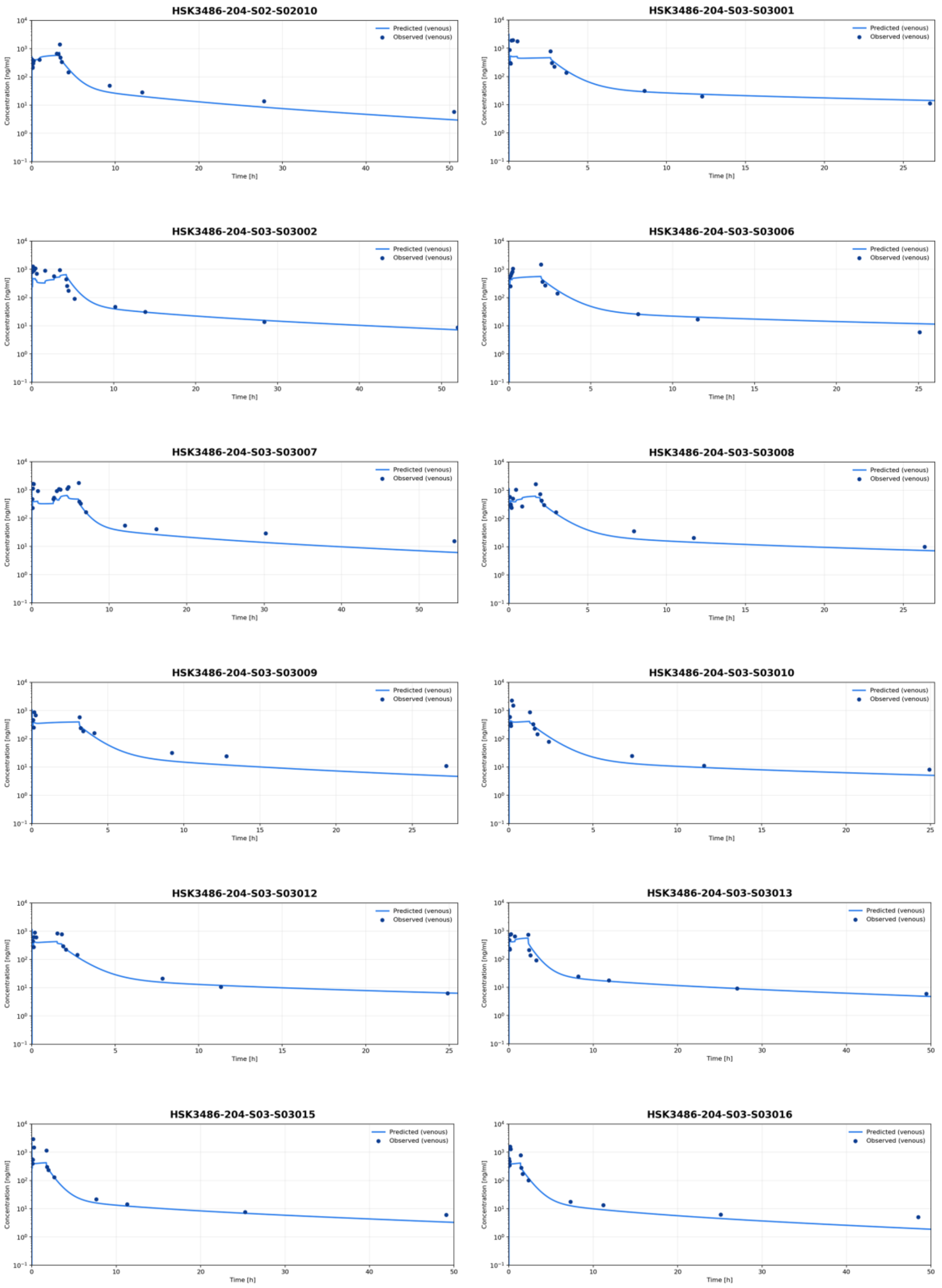

Figure S1 (continued)

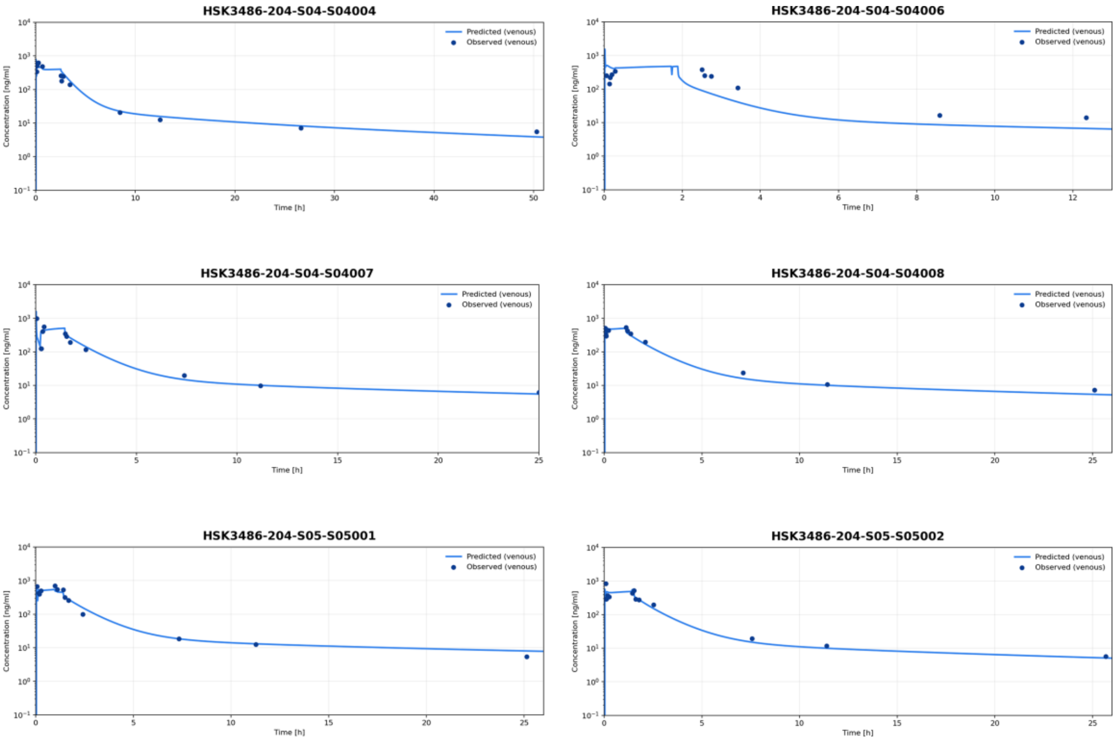

Figure S1 (continued)

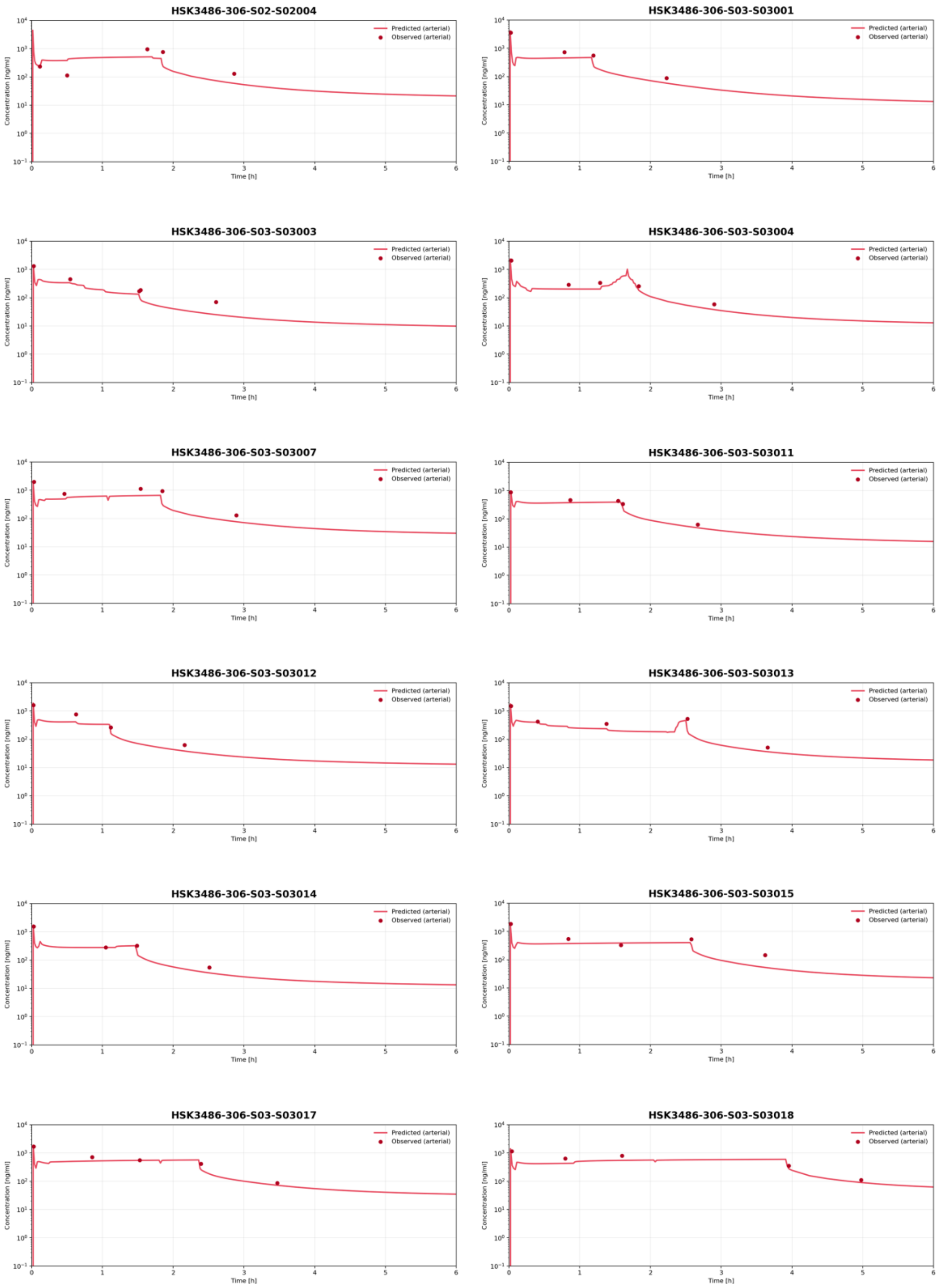

Figure S2 Predicted and observed arterial plasma concentration-time profiles of cipepofol in individual patients from HSK3486-306.

Each subpanel represents one patient. The solid red lines represent model-predicted arterial plasma concentrations, and the dark red symbols represent the corresponding observed arterial plasma concentrations.

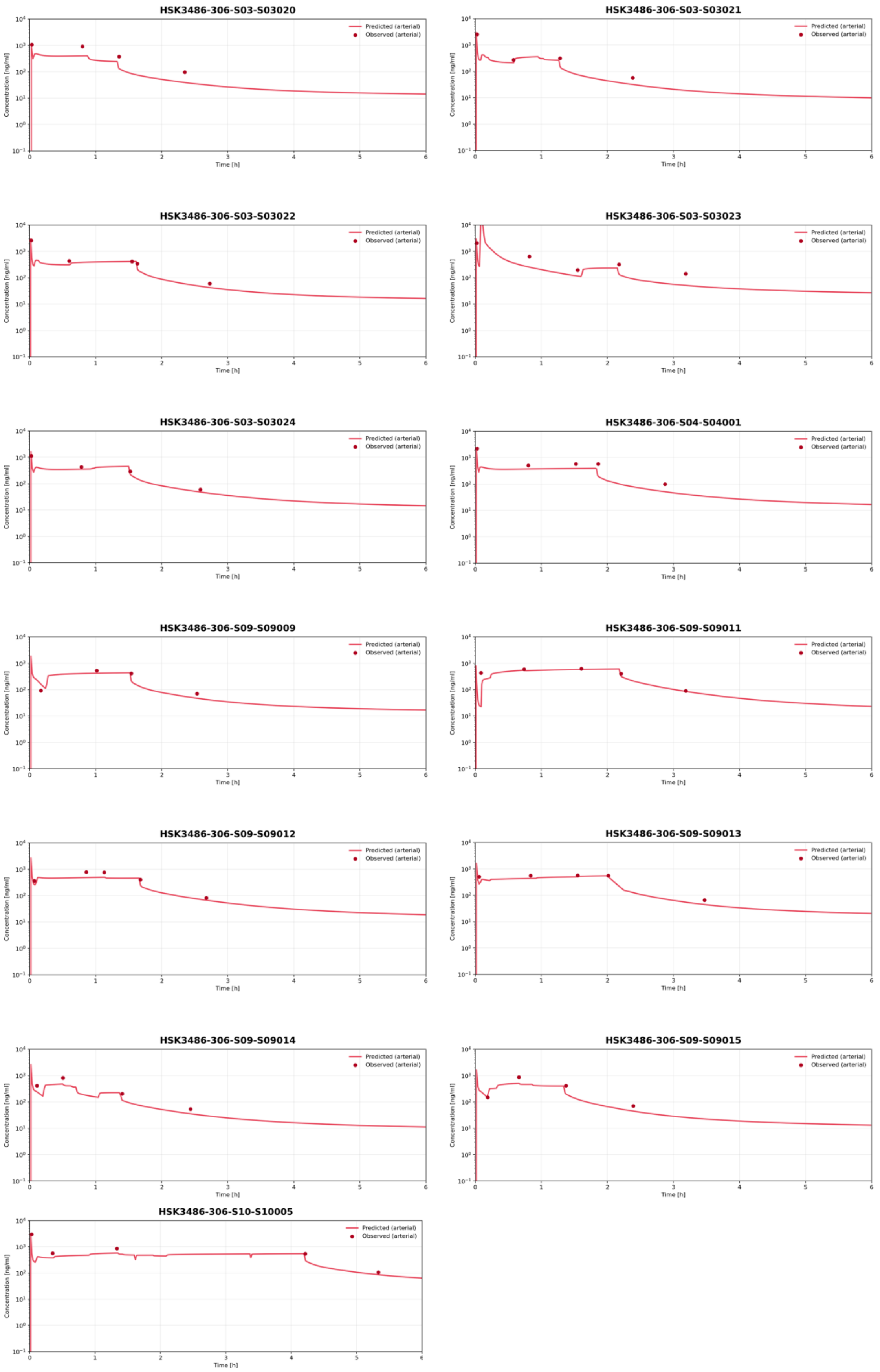

Figure S2 (continued)

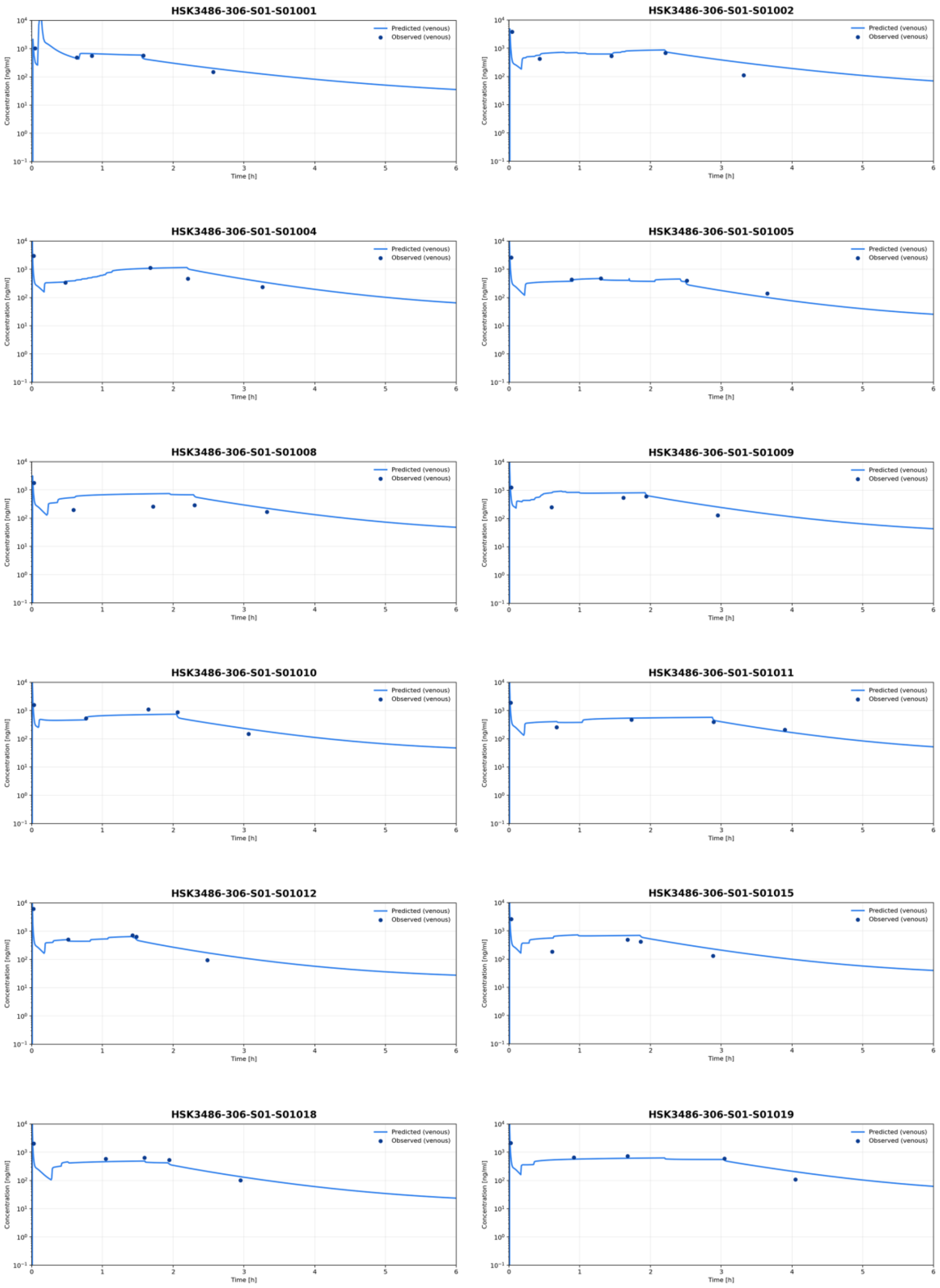

Figure S3 Predicted and observed venous plasma concentration-time profiles of cipepofol in individual patients from HSK3486-306.

Each subpanel represents one patient. The solid blue lines represent model-predicted venous plasma concentrations, and the dark blue symbols represent the corresponding observed venous plasma concentrations.

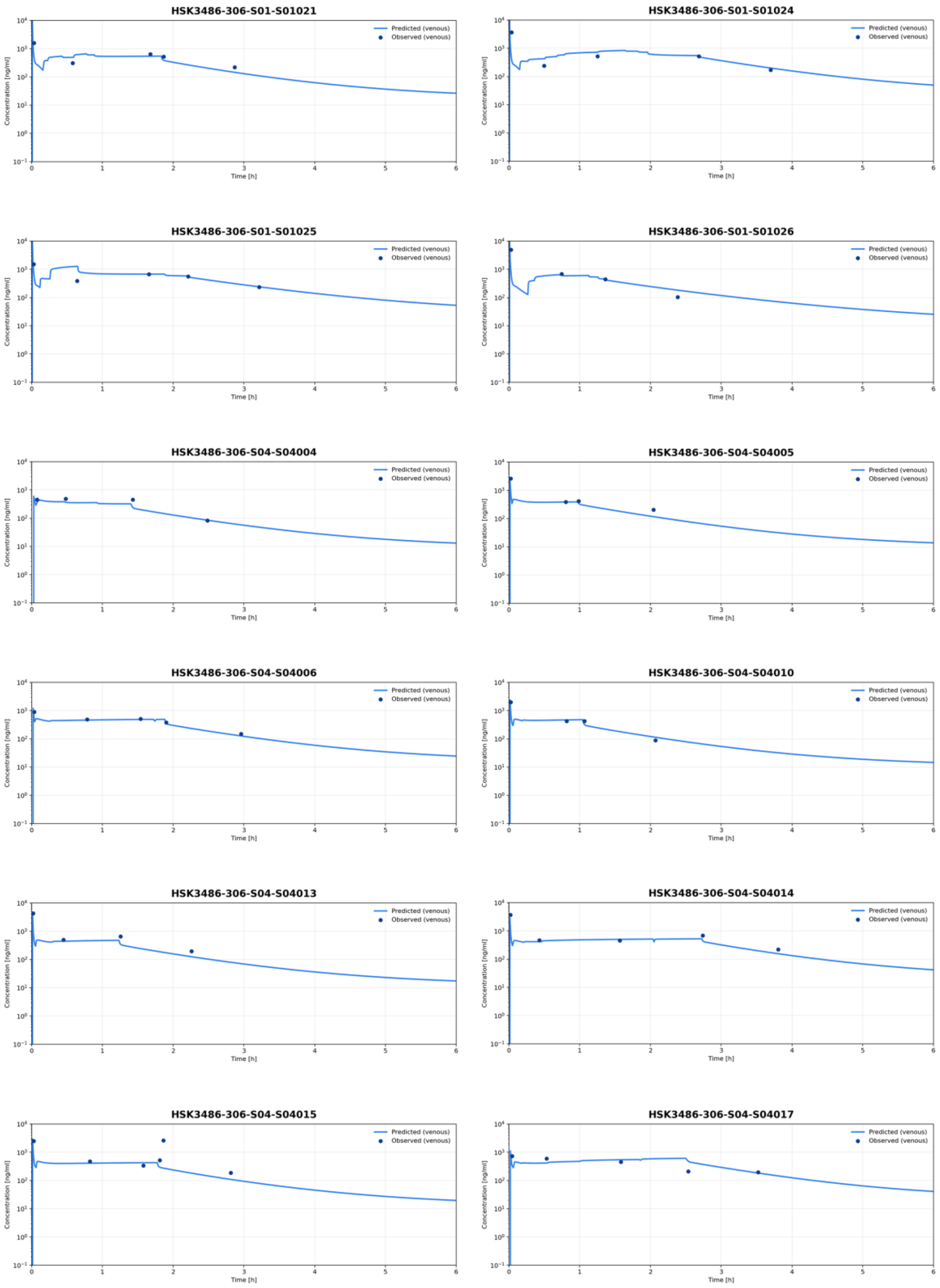

Figure S3 (continued)

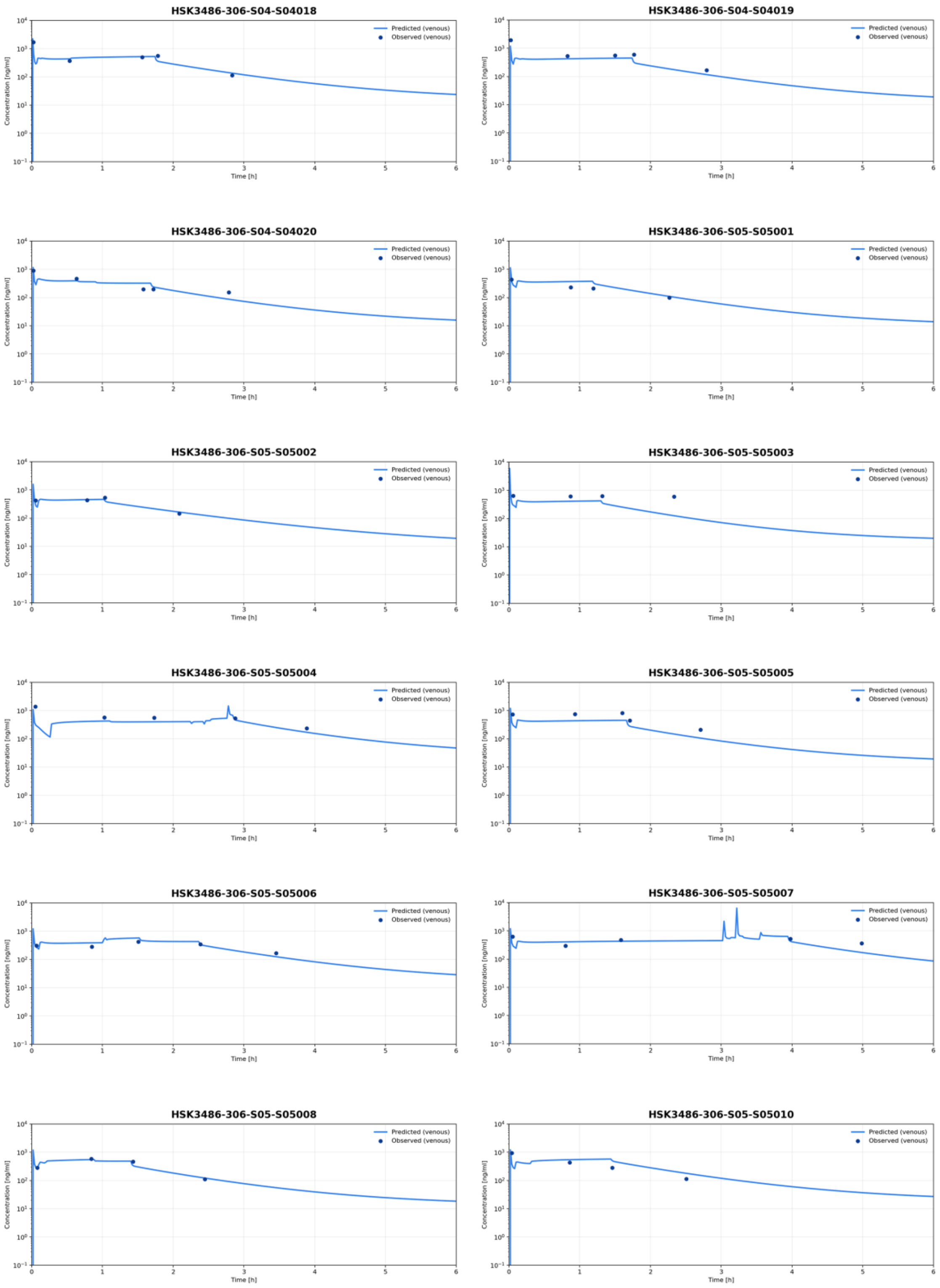

Figure S3 (continued)

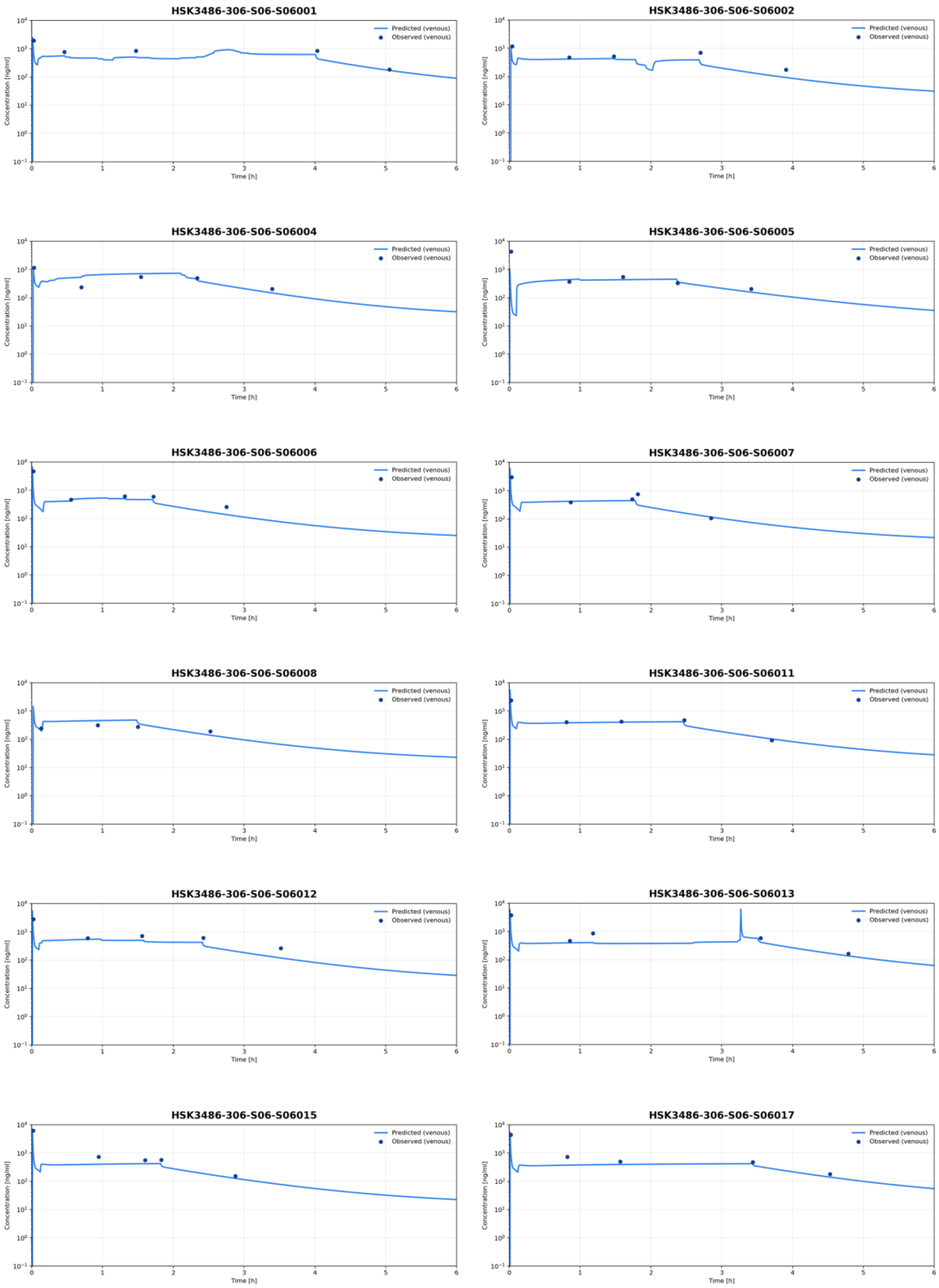

Figure S3 (continued)

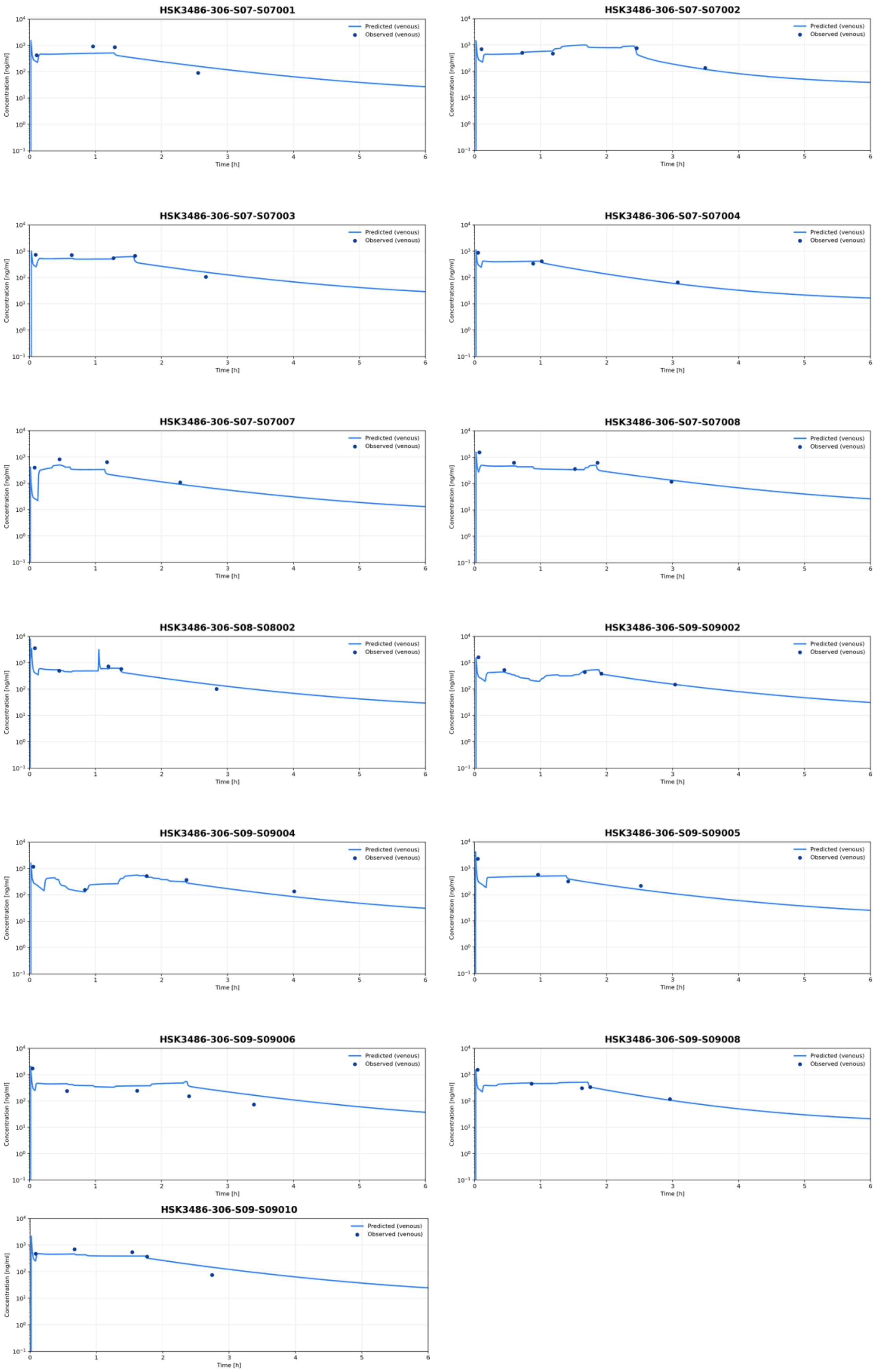

Figure S3 (continued)

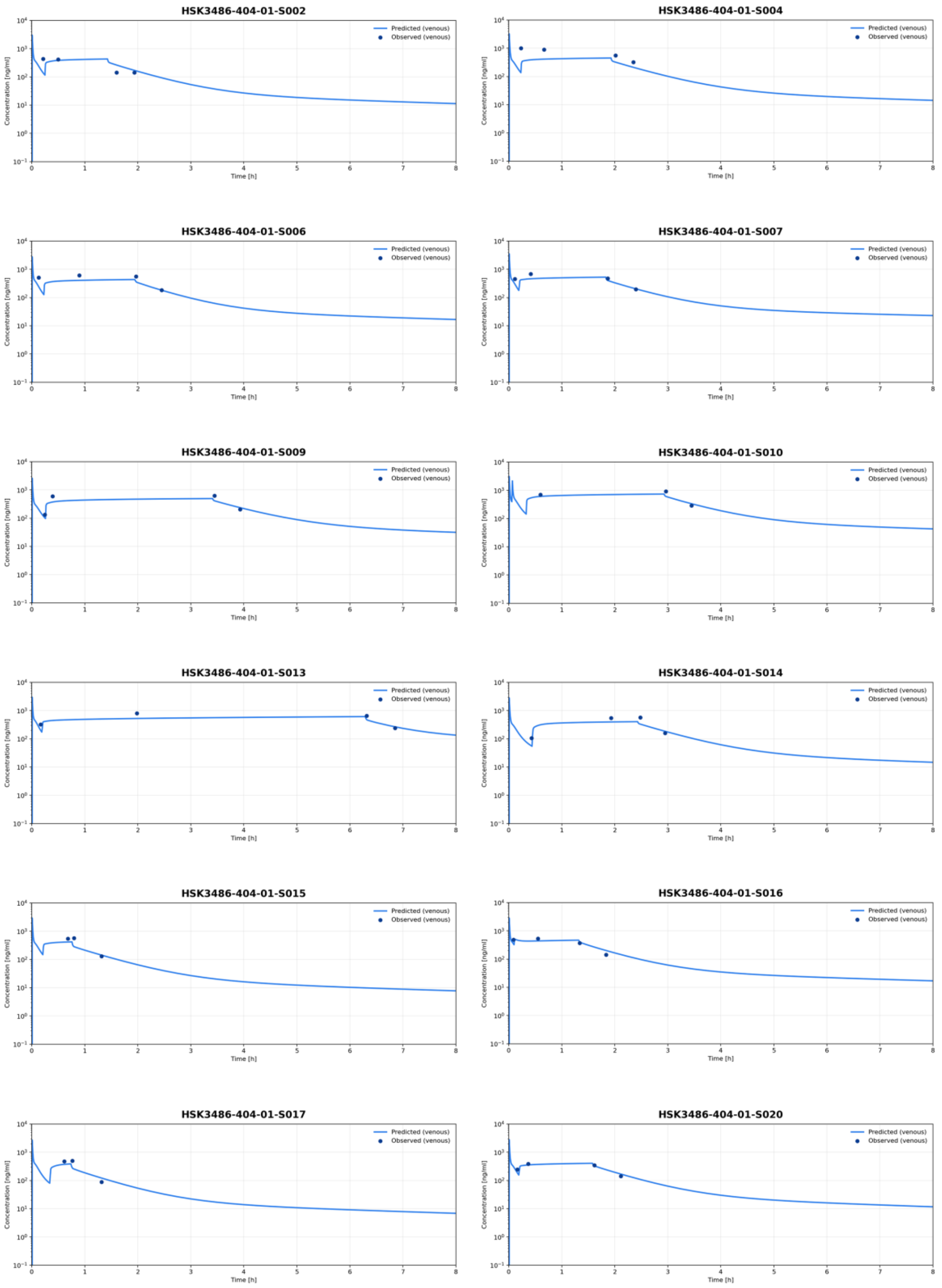

Figure S4 Predicted and observed venous plasma concentration-time profiles of cipepofol in individual pediatric patients aged 2-5 years from HSK3486-404.

Each subpanel represents one patient. The solid blue lines represent model-predicted venous plasma concentrations, and the dark blue symbols represent the corresponding observed venous plasma concentrations.

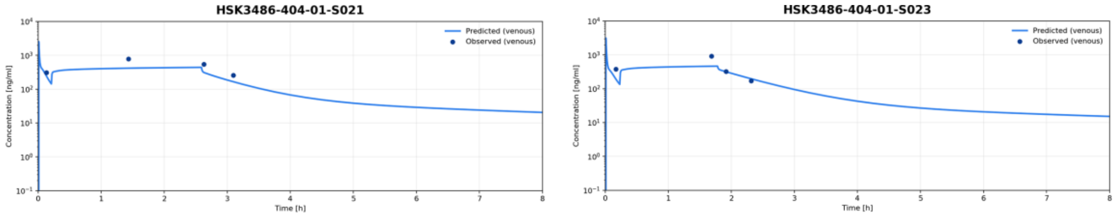

Figure S4 (continued)

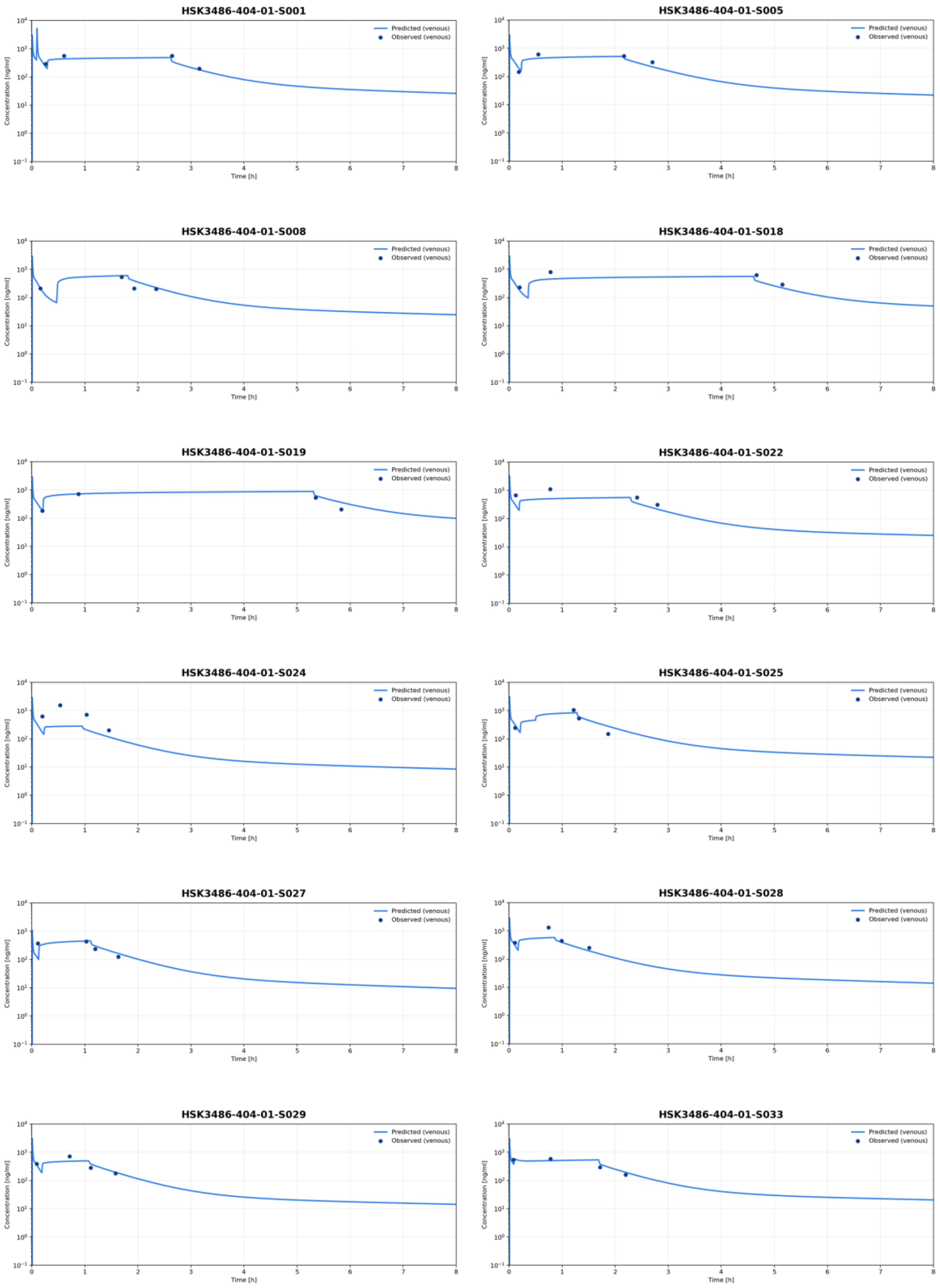

Figure S5 Predicted and observed venous plasma concentration-time profiles of cipepofol in individual pediatric patients aged 6-11 years from HSK3486-404. Each subpanel represents one patient. The solid blue lines represent model-predicted venous plasma concentrations, and the dark blue symbols represent the corresponding observed venous plasma concentrations.

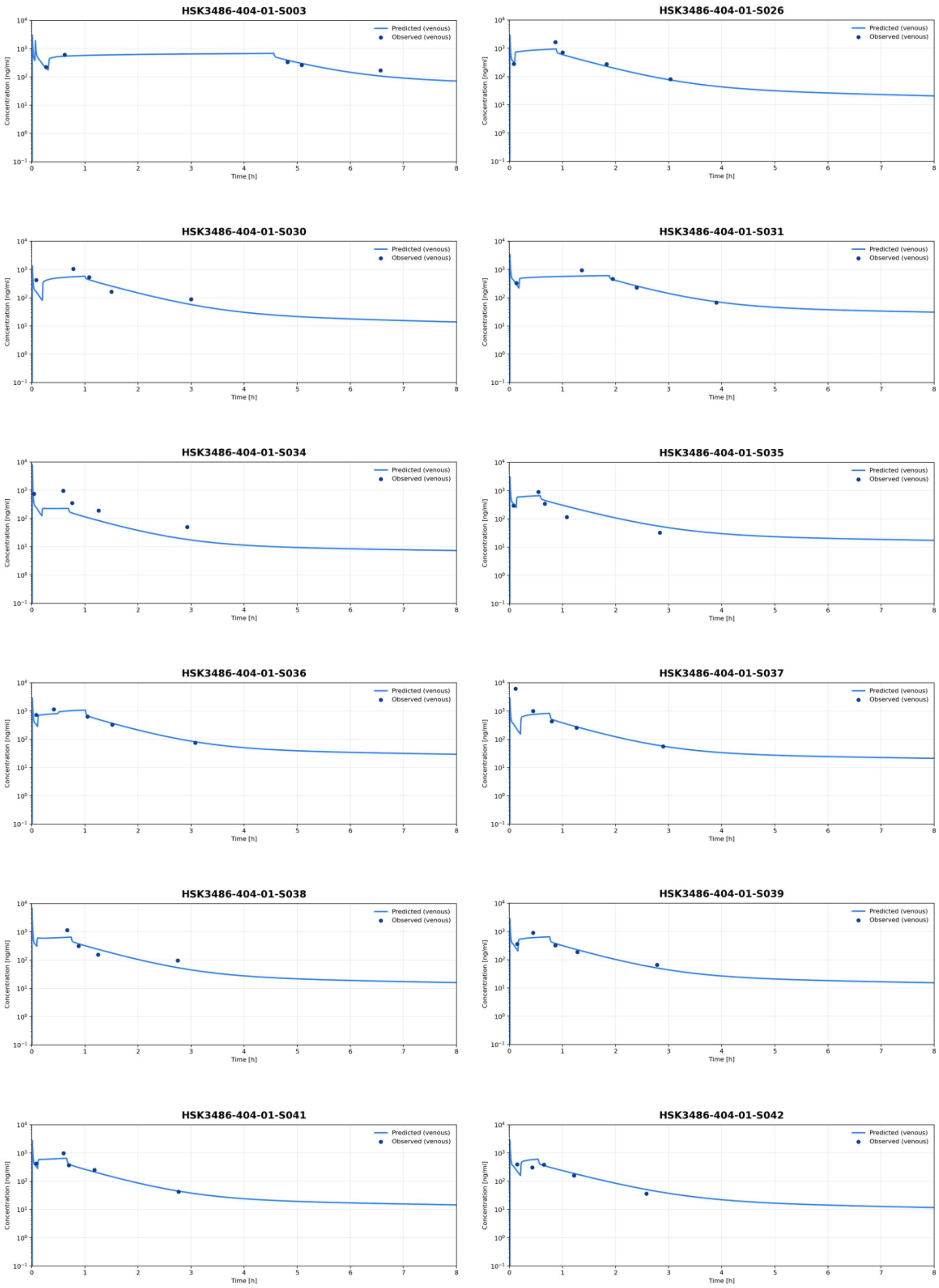

Figure S6 Predicted and observed venous plasma concentration-time profiles of cipepofol in individual pediatric patients aged 12-17 years from HSK3486-404. Each subpanel represents one patient. The solid blue lines represent model-predicted venous plasma concentrations, and the dark blue symbols represent the corresponding observed venous plasma concentrations.

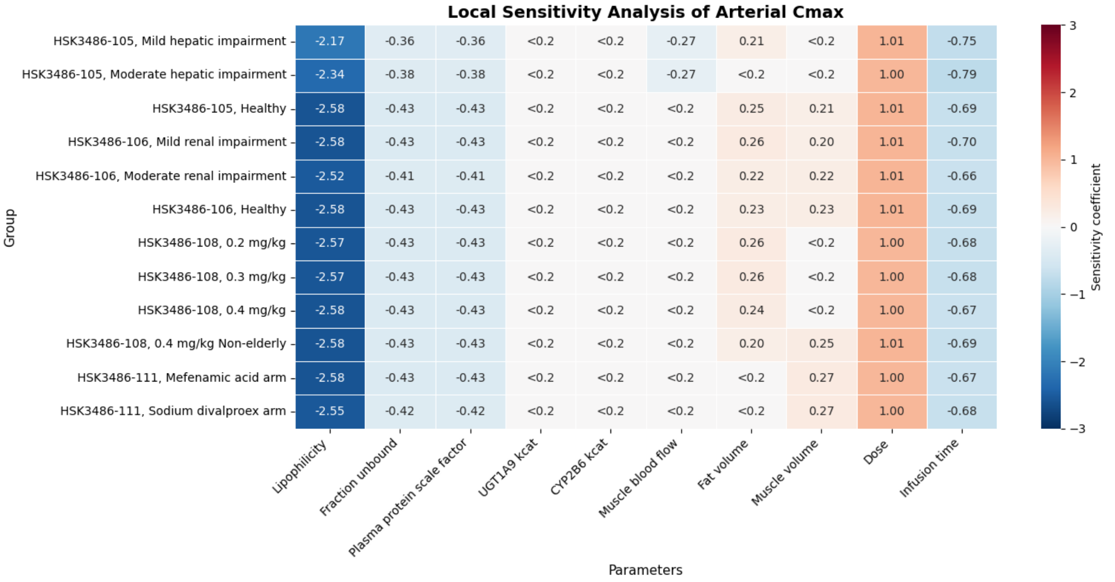

Figure S7 Local sensitivity analysis of arterial C_{max} across clinical populations and dosing scenarios.

Sensitivity coefficients of arterial C_{max} to key drug-specific, physiological, metabolic, and dosing-related parameters are shown across multiple clinical populations. Arterial C_{max} was most sensitive to cipepofol lipophilicity, dose, and infusion duration. Plasma protein binding-related parameters showed moderate effects, whereas physiological distribution- and metabolic enzyme-related parameters exhibited low influences. Positive values indicate increased arterial C_{max} with increasing parameter values, while negative values indicate the opposite. Sensitivity coefficients with absolute values <0.2 were considered to have negligible influence and are displayed as "<0.2".

Supplementary References

17. Annet, L.; Materne, R.; Danse, E.; Jamart, J.; Horsmans, Y.; Van Beers, B.E. Hepatic flow parameters measured with MR imaging and Doppler US: correlations with degree of cirrhosis and portal hypertension. *Radiology* **2003**, *229*, 409-414, doi:10.1148/radiol.2292021128.
18. Dincer, D.; Besisk, F.; Demirkol, O.; Demir, K.; Kaymakoglu, S.; Cakaloglu, Y.; Okten, A. Relationships between hemodynamic alterations and Child-Pugh Score in patients with cirrhosis. *Hepatogastroenterology* **2005**, *52*, 1521-1525.
19. Edginton, A.N.; Willmann, S. Physiology-based simulations of a pathological condition: prediction of pharmacokinetics in patients with liver cirrhosis. *Clin Pharmacokinet* **2008**, *47*, 743-752, doi:10.2165/00003088-200847110-00005.
20. Proulx, N.L.; Akbari, A.; Garg, A.X.; Rostom, A.; Jaffey, J.; Clark, H.D. Measured creatinine clearance from timed urine collections substantially overestimates glomerular filtration rate in patients with liver cirrhosis: a systematic review and individual patient meta-analysis. *Nephrol Dial Transplant* **2005**, *20*, 1617-1622, doi:10.1093/ndt/gfh839.
21. Sansoè, G.; Ferrari, A.; Castellana, C.N.; Bonardi, L.; Villa, E.; Manenti, F. Cimetidine administration and tubular creatinine secretion in patients with compensated cirrhosis. *Clin Sci (Lond)* **2002**, *102*, 91-98.
22. Woitas, R.P.; Stoffel-Wagner, B.; Flommersfeld, S.; Poege, U.; Schiedermaier, P.; Klehr, H.U.; Spengler, U.; Bidlingmaier, F.; Sauerbruch, T. Correlation of serum concentrations of cystatin C and creatinine to inulin clearance in liver cirrhosis. *Clin Chem* **2000**, *46*, 712-715.
23. Wong, F.; Girgrah, N.; Graba, J.; Allidina, Y.; Liu, P.; Blendis, L. The cardiac response to exercise in cirrhosis. *Gut* **2001**, *49*, 268-275, doi:10.1136/gut.49.2.268.
24. Barry, M.; Keeling, P.W.; Weir, D.; Feely, J. Severity of cirrhosis and the relationship of alpha 1-acid glycoprotein concentration to plasma protein binding of lidocaine. *Clin Pharmacol Ther* **1990**, *47*, 366-370, doi:10.1038/clpt.1990.41.
25. Johnson, T.N.; Boussery, K.; Rowland-Yeo, K.; Tucker, G.T.; Rostami-Hodjegan, A. A semi-mechanistic model to predict the effects of liver cirrhosis on drug clearance. *Clin Pharmacokinet* **2010**, *49*, 189-206, doi:10.2165/11318160-000000000-00000.
26. Edginton, A.N.; Schmitt, W.; Willmann, S. Development and evaluation of a generic physiologically based pharmacokinetic model for children. *Clin Pharmacokinet* **2006**, *45*, 1013-1034, doi:10.2165/00003088-200645100-00005.
27. Malik, P.R.V.; Yeung, C.H.T.; Ismaeil, S.; Advani, U.; Djie, S.; Edginton, A.N. A Physiological Approach to Pharmacokinetics in Chronic Kidney Disease. *J Clin Pharmacol* **2020**, *60 Suppl 1*, S52-s62, doi:10.1002/jcph.1713.
28. Hsu, C.Y.; Bates, D.W.; Kuperman, G.J.; Curhan, G.C. Relationship between hematocrit and renal function in men and women. *Kidney Int* **2001**, *59*, 725-731, doi:10.1046/j.1523-1755.2001.059002725.x.
